# Monomers, Dimers, and Oligomers of Pyroglutamate-Modified α‑Synuclein Fragments Exhibit Distinct Biophysical Characteristics

**DOI:** 10.1021/acschemneuro.5c00106

**Published:** 2025-04-30

**Authors:** Alexandra Bluhm, Wei Xiang, Frank Wien, Aurelien Thureau, Maelenn Chevreuil, Bertrand Raynal, Stefanie Geissler, Michael Wermann, Stephan Schilling, Philippe Bénas, Maike Hartlage-Rübsamen, Anja Schulze, Claude Sauter, Steffen Roßner

**Affiliations:** † Paul Flechsig Institute − Centre for Neuropathology and Brain Research, University of Leipzig, 04103 Leipzig, Germany; ‡ University Hospital Erlangen, Department Molecular Neurology, Friedrich-Alexander-University Erlangen-Nürnberg, 91054 Erlangen, Germany; § Synchrotron SOLEIL, L’Orme des Merisiers Saint Aubin, 91410 Gif-sur-Yvette, France; ∥ Plateforme de biophysique moléculaire, C2RT, Institut Pasteur, Université Paris Cité, 75015 Paris, France; ⊥ 28433 Fraunhofer Institute for Cell Therapy and Immunology, Department of Molecular Drug Design and Target Validation, 06120 Halle (Saale), Germany; # Faculty of Applied Biosciences and Process Engineering, Anhalt University of Applied Sciences, 06366 Köthen, Germany; ∇ CNRS, Architecture et Réactivité de l’ARN, UPR 9002, Institut de Biologie Moléculaire et Cellulaire, Université de Strasbourg, 67084 Strasbourg, France

**Keywords:** α-synuclein, N-terminal truncation, pyroglutamate modification, biophysical characterization, EOM modeling

## Abstract

α-Synuclein (aSyn) aggregation represents a key event in the neurodegenerative cascade of synucleinopathies. Initially, aSyn appears as an intrinsically disordered protein. However, its structural flexibility allows aSyn to either adopt α-helical conformations, relevant for physiological functions at presynaptic vesicles, or form β-strand-rich aggregates, leading to toxic oligomers. This relation between structure, function, and toxicity can be influenced by post-translational modifications such as the recently identified glutaminyl cyclase-catalyzed pyroglutamate (pE) modification. Here, we investigated (i) structural characteristics of monomeric, dimeric, and oligomeric states of N-terminal truncated, pE-modified aSyn variants, pE24-, pE62-, and pE79-aSyn by a complementary biophysical approach including DLS, SEC-MALS, SRCD, SEC-SAXS, and AUC and (ii) the toxicity of oligomeric pE-aSyn variants compared to full-length aSyn. Overall, pE62-aSyn showed an immediate fibril formation, reflecting the aggregation-prone properties of this particular variant. Furthermore, in a membrane-like environment, the secondary aSyn structure shifted toward α-helical folding depending on the degree of N-terminal truncation. pE79-aSyn showed a significantly reduced level of structural adaptation, reflecting compromised functions at presynaptic vesicles. In addition, the comparative analysis indicates the presence of a dimeric aSyn intermediate, the initial and potentially crucial step in aSyn aggregation, and supports the hypothesis of a toxic porous oligomeric state. For the first time, based on SAXS data, EOM models of the dimeric aSyn state are proposed.

## Graphical abstract

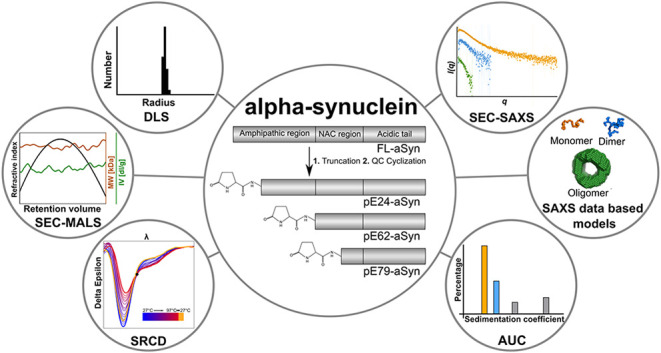

## Introduction

The protein α-synuclein (aSyn) is implicated in pathological protein aggregation in synucleinopathies, including Parkinson’s disease (PD), dementia with Lewy bodies (DLB), and multiple system atrophy (MSA).^1–5^ While in PD and DLB aSyn typically accumulates in Lewy bodies and Lewy neurites in neuronal cells, MSA is characterized by aSyn accumulation as glial cytoplasmic inclusions in oligodendrocytes.^1,3–5^ The formation of oligomeric aSyn assemblies appears to be associated with neurotoxicity.^6^ However, a clear understanding of the formation and structure of aSyn oligomers, including the initial step of dimer formation, is lacking.^7^ The aSyn primary protein structure provides a basis for understanding its multiple roles in physiology and pathology and, in particular, in aggregation processes. aSyn, with 140 amino acid (aa) residues, can be divided into three distinct regions: an amphipathic N-terminal region (aa 1–60), a hydrophobic nonamyloid-β component (NAC) region (aa 61–95), and a highly acidic, proline-rich C-terminal region (aa 96–140).^8^

In its native state, aSyn is soluble and lacks a defined secondary structure, appearing as an intrinsically disordered protein (IDP).^9,10^ However, upon membrane interaction of the N-terminus, aSyn can adopt an α-helical conformation, which appears to play a critical role in its physiological functions.^11^ The exact function of aSyn is intensively discussed in the field. For example, the specific transport of aSyn to presynaptic terminals indicates a role in synaptic function and plasticity.^12–14^ Burré *et al.* (2010) shed light on the involvement of aSyn in neurotransmitter release by supporting SNARE complex assembly via binding to Synaptobrevin-2.^15^ However, as the name **synucle**in implies, the protein was detected not only in **syn**apses but also in the **nucl**eus. Evidence suggests that aSyn can translocate to the nucleus via active transport mechanisms and interact with nuclear components, including DNA, histones, and transcription factors.^16–20^ Besides the described physiological functions, aSyn is also implicated in pathological processes.^5^ Specifically, through interchain interactions of the aSyn NAC region, the conformation can adapt to highly ordered β-strand-rich structures, leading to the formation of toxic oligomers and fibrils that disturb physiological processes and are thus hallmarks of synucleinopathies.^21^

Villar-Piqué *et al.* (2016) described the complex relation between structure, function, and toxicity in a symbolic “aSyn Bermuda triangle”.^2^ ,^22^ The exact triggers of aSyn misfolding, which shift the triangle from physiological function toward pathology, are still not fully understood. However, in familial PD the structural transition can be influenced by aSyn mutations such as A30P, E46K, and A53T.^23–25^ Nevertheless, most PD cases are sporadic and do not have genetic predisposition. Therefore, environmental factors^26–28^ and/or post-translational modifications (PTM) of aSyn can trigger the misfolding.

For a small protein of 140 aa, aSyn can undergo an extensive range of PTMs, including phosphorylation, ubiquitination, nitration, acetylation, and truncation, among others.^29–31^ In addition, proteolytic cleavage of aSyn remains a significant but yet underestimated modification, which may result in a loss of function of the maternal full-length (FL) protein and in additional physiological or pathological functions of the fragments generated. The proteolytic aSyn cleavage occurs in health and disease.^32–35^ C-terminal truncations by defined protease activities, such as calpains and 20S proteasome have been linked to increased aggregation propensity and fibril formation.^30,34,36–38^ Furthermore, N-terminally truncated aSyn fragments can be generated by matrix metalloproteinases,^37,39,40^ Calpain-1,^41^ Trypsin^42^ among others^32^ and may serve as substrates for further PTMs.

There are, for example, truncated aSyn peptides with an N-terminal glutamine residue such as Q24-aSyn, Q62-aSyn, and Q79-aSyn generated by defined protease activities.^32^ These fragments are potential substrates for subsequent pyroglutamate (pE) modification catalyzed by glutaminyl cyclase (QC), an enzyme, which cyclizes N-terminal glutamine and glutamate residues, respectively.^43,44^ In the pathological condition of Alzheimer’s disease (AD), the QC-catalyzed pE modification of N-truncated Abeta peptides has been shown to be highly pathogenic, promoting Abeta oligomerization and serving as the seed for plaque formation.^45–48^

Previously, we have demonstrated for the first time that QC can catalyze the cyclization of an aSyn fragment, namely Q79-aSyn, into pE79-aSyn.^49^ This variant has been detected in human PD and DLB post mortem brain tissue as well as in the brains of PD mouse models. It was found to be colocalized to the enzymes required for N-terminal aSyn truncation, namely, matrix metalloproteinase-3, and QC for subsequent pE cyclization.^40,49^ We hypothesize that other N-terminal truncated aSyn fragments, namely, pE24-aSyn and pE62-aSyn, may also play a role in aSyn aggregation.

Given the specific pathogenic profiles of oligomeric protein aggregates, we here investigated the structural properties of monomeric, dimeric, and oligomeric pE24-aSyn, pE62-aSyn, and pE79-aSyn in comparison to FL-aSyn by synchrotron radiation circular dichroism (SRCD), size exclusion chromatography coupled to multiangle light scattering (SEC-MALS) and to small-angle X-ray scattering (SEC-SAXS), as well as by analytical ultracentrifugation (AUC). It is noteworthy that our findings indicate the presence of a dimeric aSyn intermediate. The formation of dimers represents a potentially crucial step in aSyn aggregation to toxic oligomers, as indicated here by the higher toxicity of oligomeric FL-aSyn and pE-modified aSyn variants. Thus, aSyn dimers may serve as new targets for future therapies or as biomarkers in PD research.

## Results

In the present study, structural properties of monomeric and oligomeric pE24-aSyn, pE62-aSyn, and pE79-aSyn were analyzed in comparison to FL-aSyn by SRCD and SEC-SAXS, orthogonally complemented with AUC data. The quality of the samples was verified by dynamic light scattering (DLS) and SEC-MALS before proceeding with the other analytical methods. The cytotoxicity of oligomeric states of FL-aSyn and pE-aSyn variants was shown in differentiated SH-SY5Y cells.

### DLS and SEC-MALS as Quality Control of Monomeric Starting Conditions in Aggregation Assays

To ensure comparable starting conditions in the aggregation experiments, DLS measurements were carried out immediately after solubilization of the FL-aSyn and pE-aSyn variants in buffer with 20 mM Tris/HCl pH 7.2, 100 mM NaCl. Size distribution profiles from typical quality control experiments as recommended^50^ are presented in Figure 1a.

**Figure 1:**
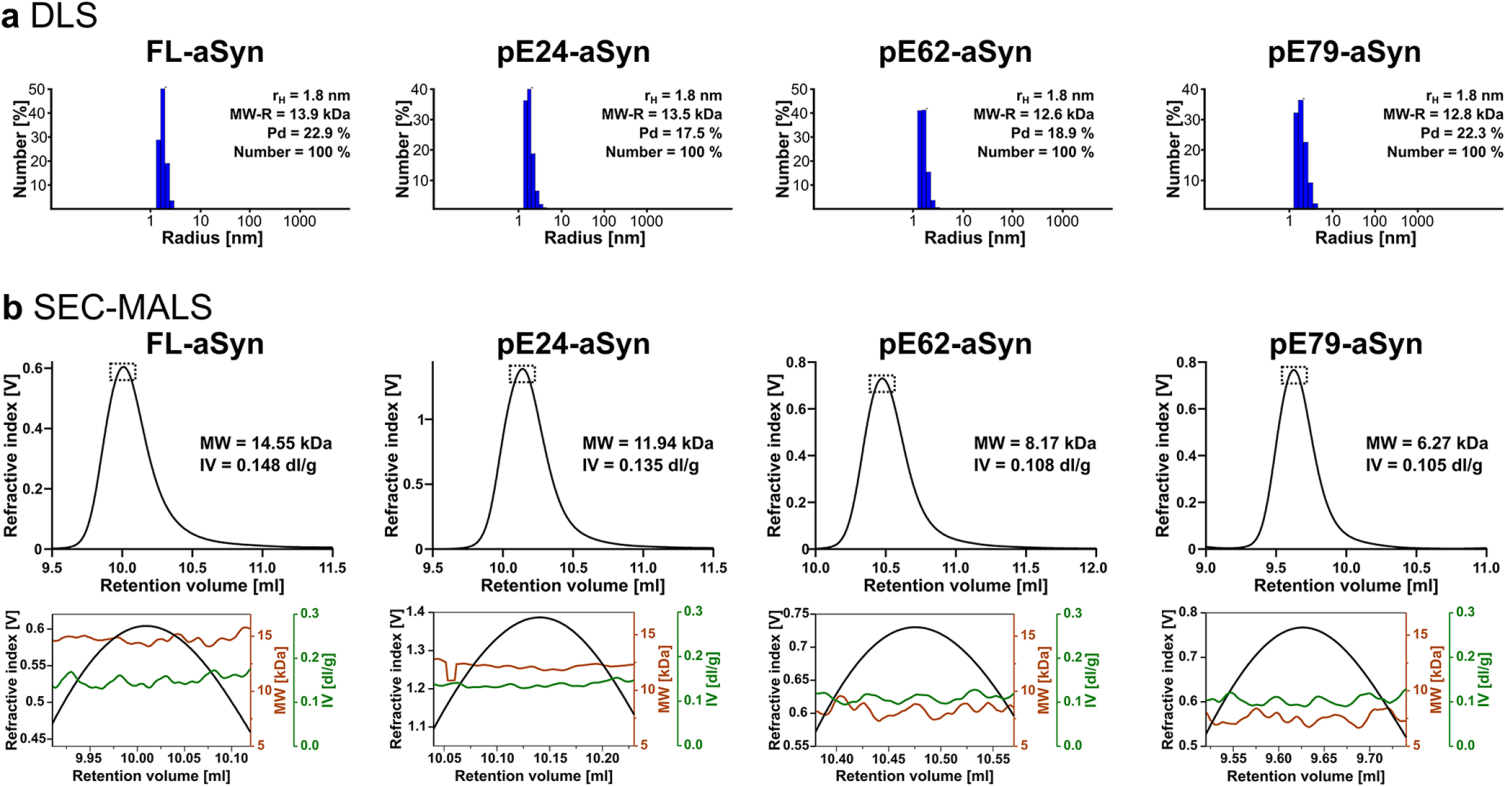
Analyses of FL-aSyn and pE-aSyn variants were performed by DLS and SEC-MALS. (a) In DLS, the number of particles was plotted as a function of the hydrodynamic radius (rH) of the particle size with peaks corresponding to the major species (numbers), the monomeric fractions of recombinant FL-aSyn and pE-aSyn variants. On each graph’s top right, the hydrodynamic parameters (rH, molecular weight (MW-R) and polydispersity (Pd)) derived from the measurements are displayed. (b) In the SEC-MALS measurements, the refractive index is plotted as a function of the retention volume. On each peak, experimentally derived parameters (MW and intrinsic viscosity [IV]) are presented (top row). In the lower figures, the corresponding peak apexes are highlighted (bottom row).

aSyn size distributions exhibited a single peak with a calculated hydrodynamic radius of 1.8 nm, which could be attributed to the monomeric species. The polydispersity ranged from 17.5% of pE24-aSyn up to 25.7% of pE79-aSyn, which is probably due to nonglobular, most likely slightly elongated and flexible shapes.^51^ The estimated molecular weights were 13.9, 13.5, 12.6, and 12.8 kDa, respectively, for FL-aSyn, pE24-aSyn, pE62-aSyn, and pE79-aSyn (Figure 1a). Taken together, these values confirmed that the FL-aSyn and pE-aSyn variants were in a monomeric state under initial conditions. These observations were verified by SEC-MALS experiments. As shown in Figure 1b, absolute molecular masses (MW) were determined at 14.6, 11.9, 8.2, and 6.3 kDa for FL-aSyn, pE24-aSyn, pE62-aSyn, and pE79-aSyn, respectively, which represent the expected value for the monomeric species. It has to be noted that the intrinsic viscosity (IV)—a parameter that gives information about the shape and the hydration of the molecule, decreased from 0.15 dL/g for FL-aSyn to 0.11 dL/g for pE79-aSyn. Its expression is the product of the viscosity increment ν and the swollen volume *V*_s_ = (*ν̅* + δ/ρ) with ν̅ being the partial specific volume (Table S2), δ the hydration (0.488 g/g), ρ the density of the buffer (Table S2), [η] = ν·(*ν̅* + δ/ρ). The viscosity increment describes the elongation of the characterized molecule: a sphere has a value of 2.5, and the value increases with elongation. Using the IV values, it could be calculated that ν decreased from 12.1 for FL-aSyn to 8.3 for pE79-aSyn, respectively. These values indicate the persistence of an elongated and an intrinsically disordered state of the proteins in solution regardless of their length.

### SRCD: Estimation of Secondary Structural Elements and Reversibility of FL-aSyn and pE-aSyn Variants in Solution

The composition of secondary structure elements of FL-aSyn and pE-aSyn variants and their reversibility were investigated by SRCD. The aSyn proteins were measured immediately after solubilization in buffer with 20 mM Tris/HCl at pH 7.2 and 100 mM NaCl. The SRCD spectra were recorded from a wavelength of 180–260 nm (Figure 2a).

**Figure 2:**
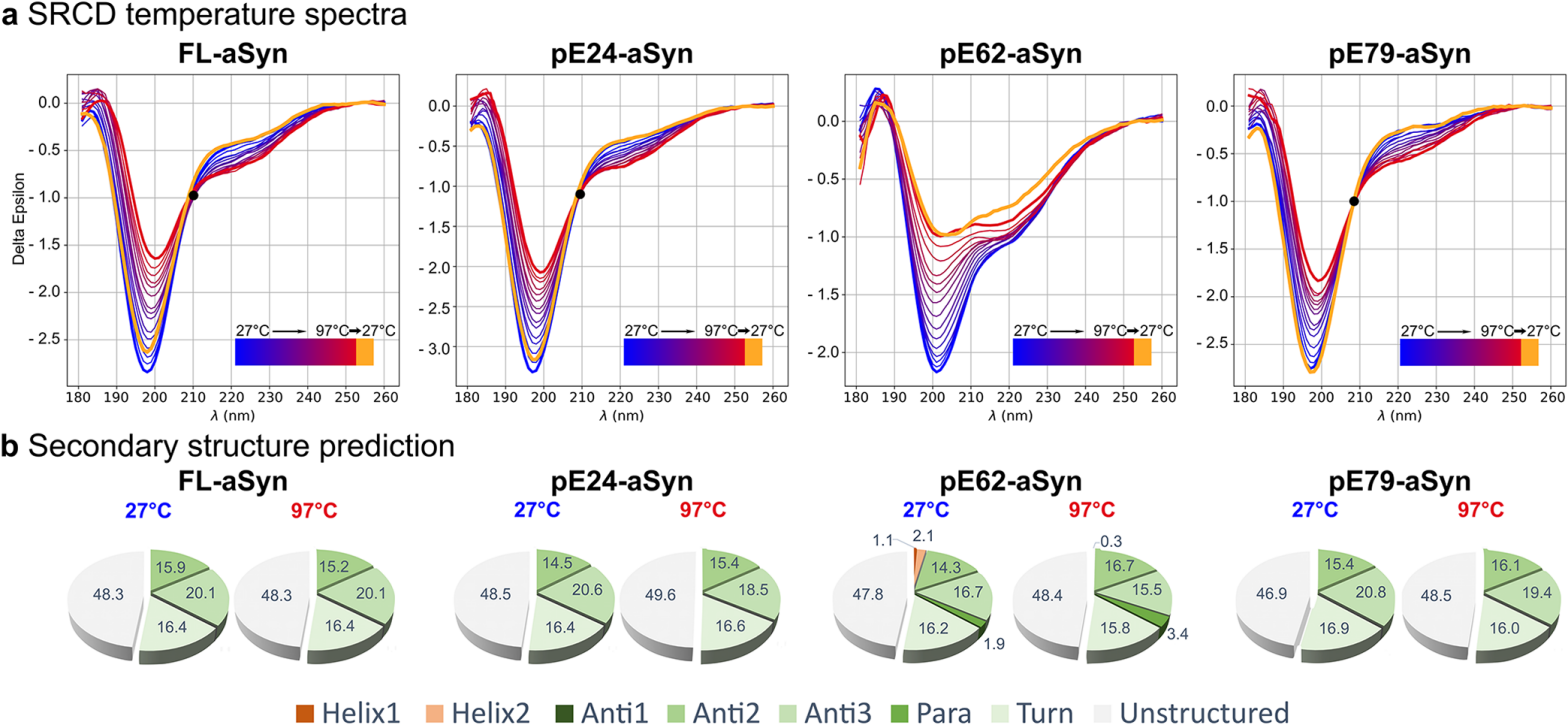
SRCD spectra profiles of FL-aSyn and pE-aSyn variants in solution within a temperature range from 27 to 97 °C and predicted secondary structure content at 27 and 97 °C. (a) SRCD spectra from 180 to 260 nm in 20 mM Tris/HCl and 100 mM NaCl buffer (pH 7.2) are characteristic of an unfolded and a random coiled structure with a single negative peak at ∼199 nm. Colors of the curves indicate different temperatures from 27 °C (blue) to 97 °C (red), in steps of 5 °C and reverse directly to 27 °C (resulting curve shown in orange). If present, an isosbestic point is marked with a black dot. (b) BESTSEL calculated proportions of secondary structure elements of FL-aSyn and pE-aSyn variants within a wavelength range of 185–250 nm at 27 and 97 °C. α-helices (red colors) are divided into regular helices with a middle part of α-helices (Helix1) and distorted helices with two-two residues at the ends (Helix2). For β-sheets (green colors), four subcategories are defined: parallel β-sheet and antiparallel β-sheet of three different twists (Para), left-hand twisted (Anti1), relaxed and slightly right-hand twisted (Anti2), and right-hand twisted (Anti3). Additionally, turns (light green) were calculated, and all other elements were declared as unstructured (gray).

For all aSyn variants, the shapes and single negative peak ∼199 nm of far-UV spectra at 27 °C (blue curves) indicated a highly unstructured IDP, which is consistent with the literature corresponding to monomeric aSyn.^52–54^ The concept of a distinct melting temperature determination (temperature at which a protein transits from folded to unfolded state) does not directly apply to the proteins investigated, which lack a well-defined structure. Nevertheless, CD spectroscopy is still a valuable tool for studying temperature-dependent conformational dynamics. Since post-translational modifications can alter the thermal stability and its reversibility, SRCD spectra of FL-aSyn and pE-aSyn variants were compared by heating the proteins gradually from 27 to 97 °C in steps of 5 °C. Subtle temperature-dependent changes in the spectra of FL-aSyn, pE24-aSyn, and pE79-aSyn were observed, such as the decrease of the negative 199 nm peak, the increase of the 222 nm band, and the increase of the peak at 185 nm. In comparison, the spectral curve of pE62-aSyn over temperature appeared to be different. Although the decrease of the negative 199 nm peak was also observed, there was an additional temperature-dependent decrease in the peak around 222 nm.

For FL-aSyn, pE24-aSyn, and pE79-aSyn, an isosbestic point at the wavelength ∼209 nm was detectable, where the total absorbance did not change (see black dotted line in Figure 2a). Isosbestic points indicate that the stoichiometry of two molecular entities remain unchanged during the temperature increase.^55^ In contrast, no isosbestic point could be identified for pE62-aSyn.

After heating the proteins to 97 °C, the samples were cooled directly to 27 °C to address the question of structural reversibility. The resulting curves are represented in Figure 2a in orange. An almost identical pattern for preheating and postheating was observed for FL-aSyn, pE24-aSyn, and pE79-aSyn, indicating that conformational plasticity is completely reversible upon temperature increase for these aSyn variants. For pE62-aSyn, however, there was an irreversible change in the postheat curve toward β-strand structures during heating. These data for pE62-aSyn in combination with a macroscopic change in sample color from transparent to opaque indicated fibrillation induced by temperature increase.

The BESTSEL secondary structure prediction (see Figure 2b) confirmed the status of unstructured IDPs with ∼50% of unstructured content at 27 °C for FL-aSyn as well as pE-variants. For all proteins investigated, comparable secondary structure contents were predicted with 0 to ∼3% α-helices, 34% β-strands, and ∼16% turns (see Figure 2b). Heated FL-aSyn and pE-aSyn variants (97 °C) showed no significant conformational changes in the secondary structure contents (see Figure 2b). The observation of reversibility was also confirmed by the same predicted secondary structure content after cooling down to 27 °C (data not shown).

### SRCD: Effect of Sodium Dodecyl Sulfate (SDS) Micelles on Folding of FL- and pE-aSyn Variants

SDS micelles were used as a membrane-mimetic environment,^56^ in order to force aSyn to structural transition and promote helical conformation. The composition of secondary structure elements of FL-aSyn and pE-aSyn variants and their reversibility upon a temperature change in the presence of SDS micelles were investigated by SRCD (see Figure 3).

**Figure 3:**
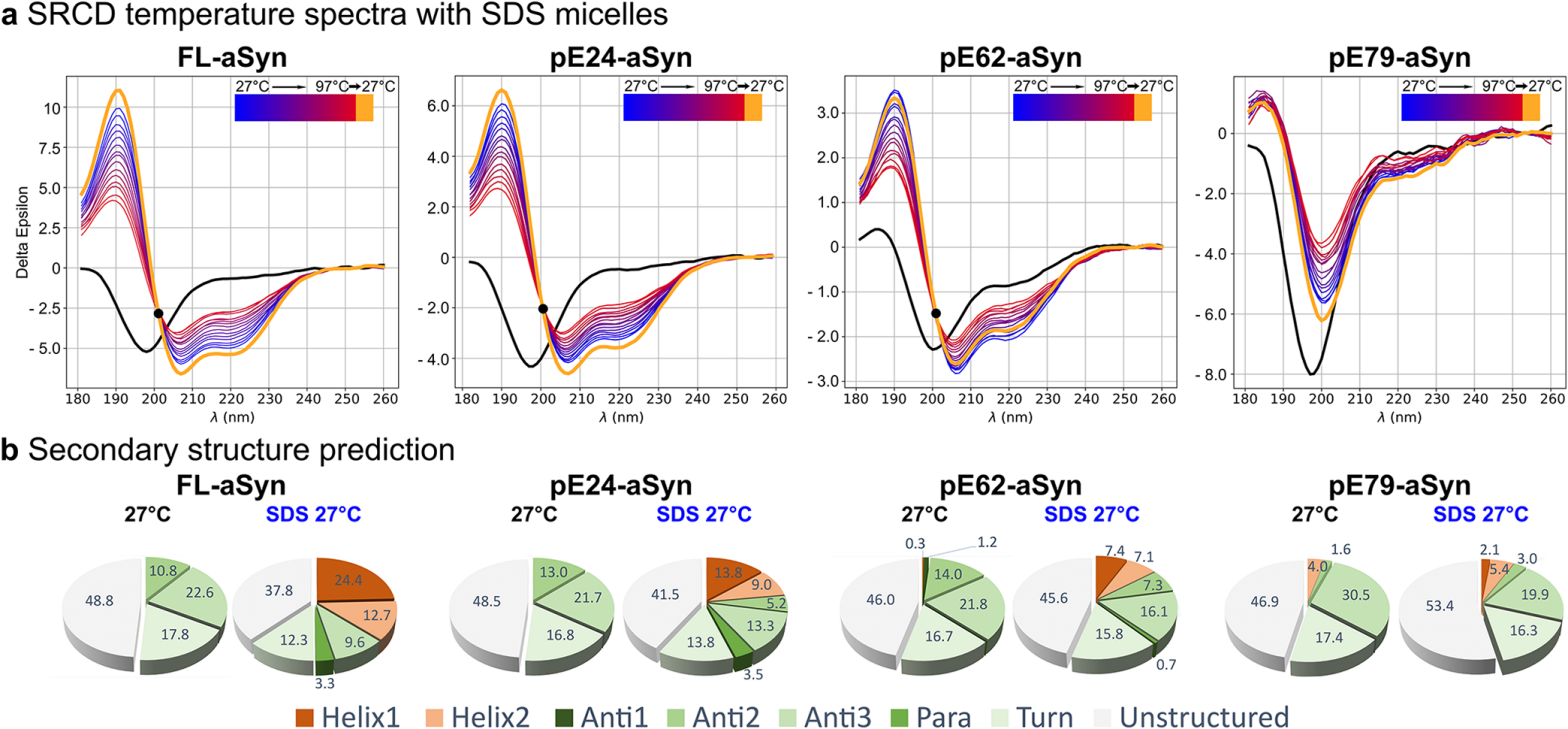
Effects of SDS micelles on SRCD spectra profiles of FL-aSyn and pE-aSyn variants within a temperature range from 27 to 97 °C and predicted secondary structure content with and without SDS micelles at 27 °C. (a) SRCD spectra from 180 to 260 nm in 20 mM Tris/HCl, 100 mM NaCl buffer (pH 7.2) without (black spectra) and with the addition of 100 mM SDS are characteristic for an α-helical structure indicated by a positive peak at 190 nm and double negative peaks at ∼208 and ∼222 nm. Colors of the curves indicate different temperatures from 27 °C (black, blue) to 97 °C (red), in steps of 5 °C and reverse directly to 27 °C (orange). If present, an isosbestic point is marked with a black dot. (b) BESTSEL calculated proportions of secondary structure elements of FL-aSyn and pE-aSyn variants within a wavelength range of 185–250 nm at 27 and 97 °C. α-helices (red colors) are divided into regular helices with a middle part of α-helices (Helix1) and distorted helices with two-two residues at the ends (Helix2). For β-sheets (green colors), four subcategories are defined: parallel β-sheet and antiparallel β-sheet of three different twists (Para), left-hand twisted (Anti1), relaxed and slightly right-hand twisted (Anti2), and right-hand twisted (Anti3). Additionally, turns (light green) were calculated, and all other elements were declared as unstructured (gray).

In order to allow direct comparison of spectra in the presence or absence of SDS, each sample was first measured without SDS (Figure 3a, black spectra), before being diluted 1:2 in the presence of 100 mM SDS and heated from 27 °C (blue) to 97 °C (red) and reversed directly to 27 °C (orange). For FL-aSyn, pE24-aSyn, and pE62-aSyn characteristic peaks for α-helices were detected; a positive peak of 190 nm with a decrease over temperature and double negative peaks ∼208 and ∼220 nm with an increase over temperature. The spectra of pE79-aSyn differed with a single negative peak ∼200 nm. Again, for FL-aSyn, pE24-aSyn, and pE62-aSyn, an isosbestic point at a wavelength of ∼200 nm was determined whereas in comparison no isosbestic point was detected for pE79-aSyn. An almost identical pattern for preheat (blue spectra) and postheat (orange spectra) was observed which indicates reversibility of all aSyn proteins investigated.

The predicted secondary structure content confirmed the capacity of SDS micelles to induce an unstructured-to-helix transition for all samples (Figure 3b). The α-helical content of FL-aSyn increased from 0 to 37.1%, that of pE24-aSyn from 0 to 22.8%, and that of pE62-aSyn from 0.3 to 14.5%, accompanied by a reduction of the β-strand and unstructured contents. Also, for pE79-aSyn, the α-helical content increased to 7.5% but unlike the other variants was accompanied by an increase in unstructured content. The reversibility of thermal transition was also confirmed by the return to a similar predicted secondary structure content after cooling down to 27 °C (data not shown).

### SEC-SAXS and AUC of Different Oligomeric States of FL-aSyn and pE-aSyn Variants

SAXS combined with SEC (for presentation of setup see Figure S1) provided information on particle size, shape, and degree of folding of different oligomeric states of FL-aSyn and pE-aSyn variants in solution. FL-aSyn and pE-aSyn variants were agitated and loaded onto an HPLC-SEC column.

Particles present in the mix were separated by size, and the 214 nm-chromatograms of the aSyn proteins collected during the SEC-SAXS experiments are shown in Figure 4a. For each sample, three peaks with different elution times were detected, except for pE62-aSyn, where only one main peak was detected. The most prominent peak, numbered p1, was eluted between 29 and 32 min, depending on the aSyn variants studied. The second peak, designated p2, was detected between 26 and 29 min, as well as a smaller peak (p3) at ∼19 min, for all aSyn variants except pE62-aSyn. Areas of the chromatograms in which SAXS signals were evaluated are indicated by color-coded boxes (p1 in orange, p2 in blue, and p3 in green). In order to gain further insight into the oligomeric states of each of the species within the peaks, several analyses were carried out using the SAXS pattern shown in Figure 4b and parameters listed in the Table S1. *R*_g_ values were evaluated by the Guinier analysis shown in Figure 4c. For FL-aSyn, *R*_g_(p1) amounted to 36.9 Å, *R*_g_(p2) to 39.5 Å, whereas *R*_g_(p3) was 64.2 Å. For pE24-aSyn, the *R*_g_ values were comparable with, *R*_g_(p1) measuring 35.0 Å, *R*_g_(p2) 48.1 Å, and *R*_g_(p3) 60.7 Å. For pE62-aSyn, the *R*_g_ value of the detected p1 was 27.9 Å. *R*_g_ values for pE79-aSyn particles *R*_g_(p1) amounted to 24.7 Å, *R*_g_(p2) to 39.8 Å, and *R*_g_(p3) to 62.6 Å, respectively, again in comparable order. Using an indirect Fourier transform of the SAXS curves, the pair-distance distribution function *P*(*r*) was calculated to determine the maximum distance *D*_max_ shown in Figure 4d as well as another estimate of *R*_g_ (for values see Table S1, *R*_g_ GNOME). In line with the decreasing elution times of the peaks p1 to p3 of FL-aSyn, *D*_max_ from p1 (orange) to p3 (green) was increasing with 145, 154, and 173 Å. The *D*_max_ for the other pE-aSyn variants showed comparable ascending values when considering p1-p3 in consistency with the lower elution times, for pE24-aSyn with 135 166, and 217 Å. For pE62-aSyn, *D*_max_ was calculated at 115 Å. For pE79-aSyn, values for p1, p2, and p3 ranged from 104 Å, 145 Å to 224 Å. The shapes and heights of p1/p2/p3 distance distribution function varied, but were comparable across variants. In Figure 4e, the dimensionless Kratky plot, which is an indicator for “globularity”, shows a hyperbolic Kratky curve that plateaus at high q values for all p1 and p2 across aSyn variants (orange and blue curves), a characteristic feature of flexible and unstructured proteins. On the contrary, Kratky curves for all p3 of aSyn variants (green curves) adopt a bell-shape indicating the presence of compactly folded particles.

**Figure 4:**
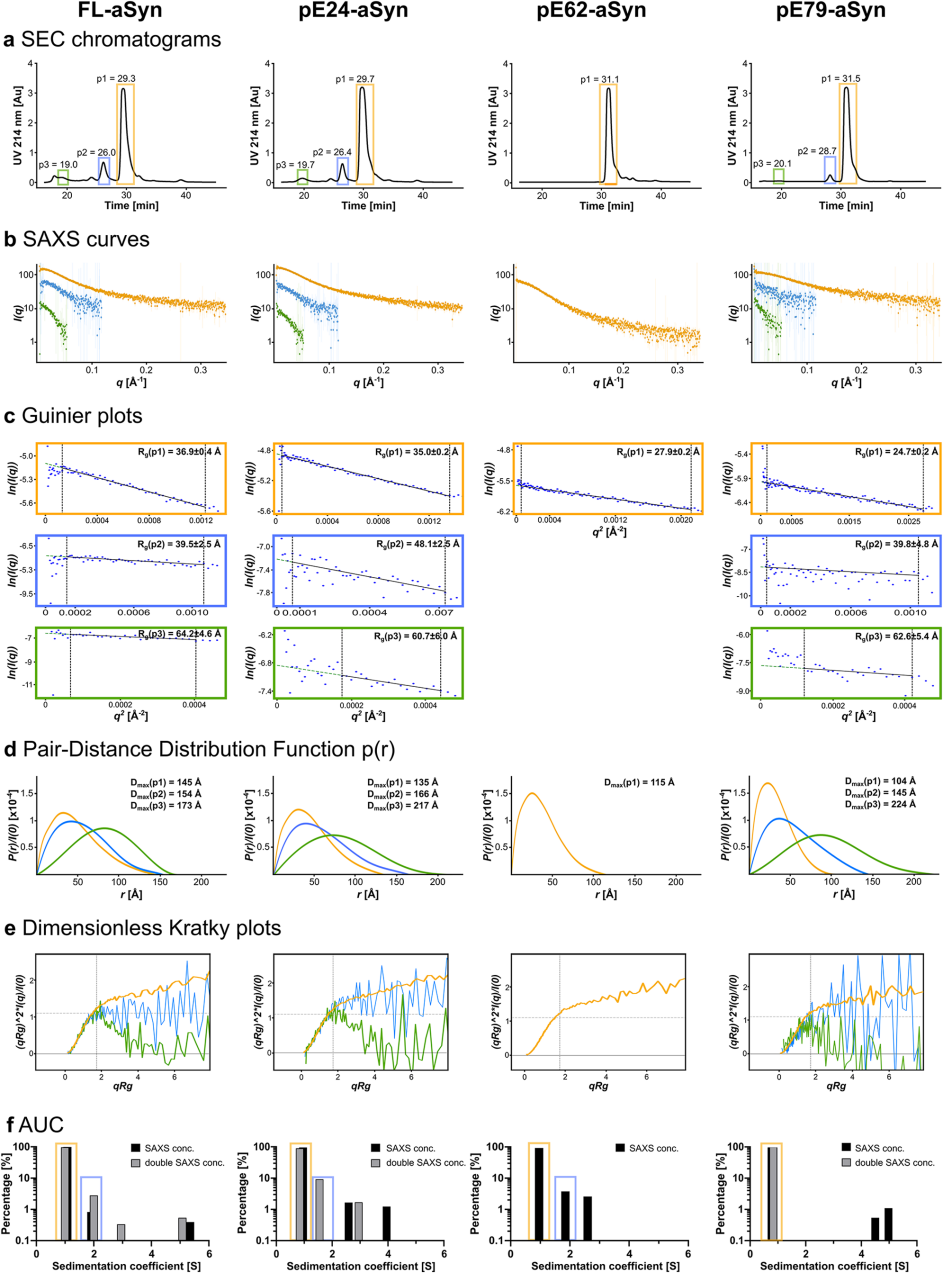
SAXS data analysis and AUC of different oligomeric states of FL-aSyn and pE-aSyn variants. (a) SEC separation after the aggregation assay was performed on a Yara3000 column at a flow rate of 0.3 mL/min, and chromatograms correspond to the absorbance recorded at 214 nm over time. The prominent peak (p1) corresponds to the monomeric fraction, while earlier peaks (p2, p3) correspond to larger particles (dimers, oligomers). The boxed areas in the chromatograms represent the areas in which SAXS signals were evaluated. The color code used throughout the figure is orange for p1 and related plots, blue for p2, and green for p3. (b) Corresponding SAXS curves of FL-aSyn and pE-aSyn variants for different oligomeric states. The scattering intensity *I*(*q*) is represented as a function of scattering vector *q*. (c) Corresponding Guinier plots calculated using BioXtas-RAW with derived radius of gyration *R*_g_ included in the top right of each plot. (d) Corresponding pair-distance distribution function *P*(*r*) calculated using GNOME, indicating maximal dimensions of the particles *D*_max_. (e) The dimensionless Kratky plots are an indicator of conformational properties of particles. The hyperbolic Kratky curve with a plateau at high *q* observed with monomers and dimers is characteristic of a disordered and flexible conformation, whereas the bell-shaped curve observed with oligomers indicates folded particles. (b–d) were plotted using BioXtas-RAW. (f) Analyses of FL-aSyn and pE-aSyn variants by analytical ultracentrifugation (AUC) were performed at two concentrations. Experimentally derived sedimentation coefficient values (Svedberg units [S]) are shown above each detected species and have a 0.2 standard deviation.

The SAXS analyses were orthogonally confirmed using AUC, an approach to study the biophysical characteristics of molecules, such as size, shape, and stoichiometry. Ultracentrifugation combined with UV/visible and interference detection systems enables hydrodynamic separation of species in solution according to their sedimentation coefficient (*S*). Using the AUC data, shown in Figure 4f, oligomeric states and corresponding hydrodynamic characteristics (*R*_H_, *f*/*f*_0_) were extracted and summarized in Table 1.

**Table 1:**
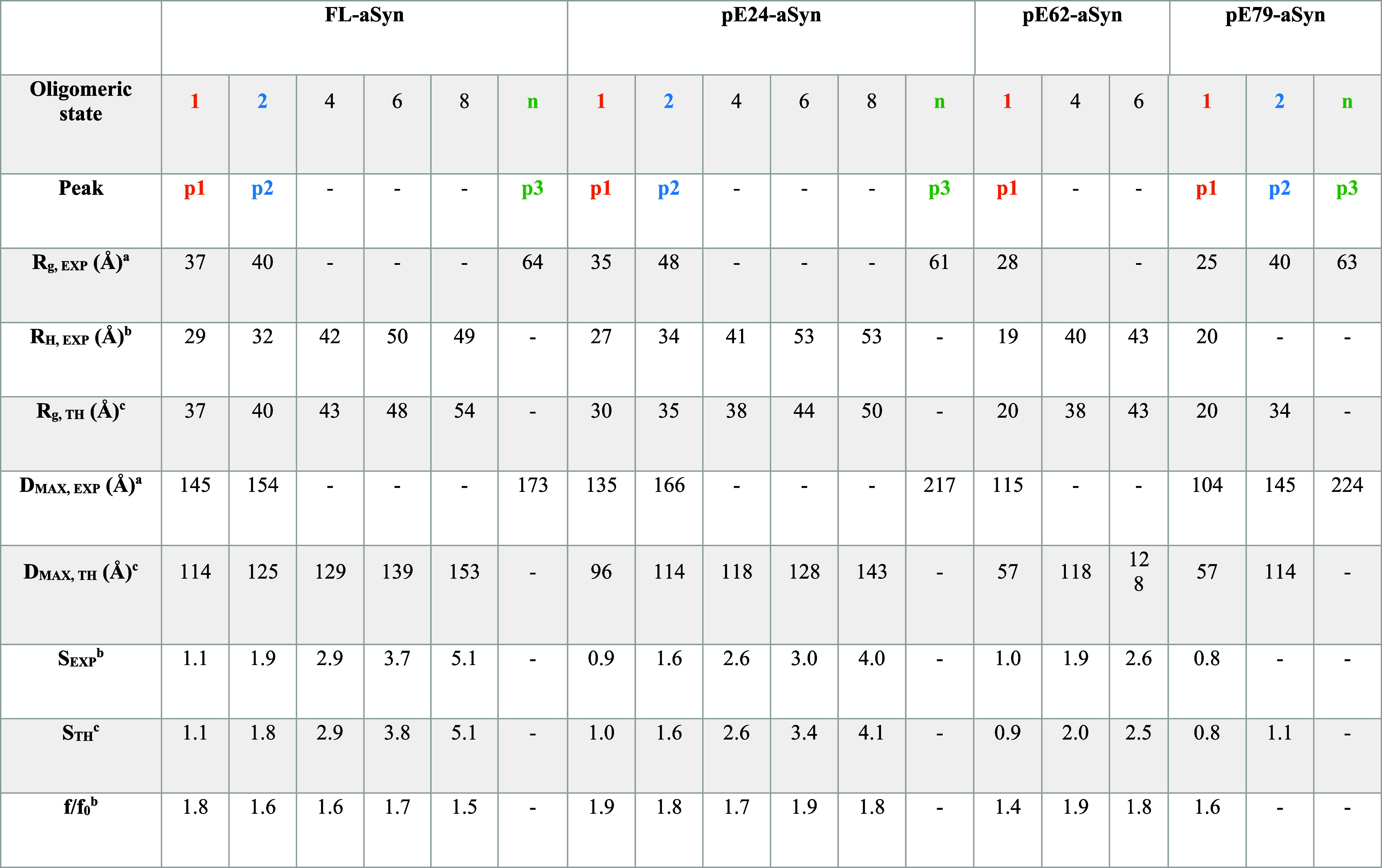
Structural and Hydrodynamic Parameters Derived from SEC-MALS, AUC, DLS, and SAXS Experiments.

Errors: *S*_EXP_: 0.1, *R*_H_,_EXP_: 1, *f*/*f*_0_: 0.1, *R*_g_, _EXP_: 0.2 to 3.

a Extracted from SAXS experimental data.

b Extracted from AUC experimental data.

c Theoretical value derived from Bloomfield model reconstruction based on SAXS and AUC data.

By linking the parameters extracted from the SAXS and AUC experiments, the main peak p1 detected for the different aSyn variants was compatible with the monomeric form of aSyn. The ratio, ρ = *R*_g_/*R*_H_, was used to get valuable information about the conformation and shape of the different aSyn variants.^57^ The typical ratio for ordered and globular proteins is expected to be around 0.7, while a ratio greater than 1 indicates more elongated/disordered conformations. The p1 peaks are characterized by a ratio of 1.3, except for the pE62-aSyn p2 species, where the ratio is 1.5, indicating an extended conformation. This observation is confirmed by the shape of the distance distribution functions, which is similar to that expected for long rods,^58^ and by the hyperbolic Kratky curves, which plateaus as q increases, indicating an unfolded particle with flexible conformation. The frictional ratio, *f*/*f*_0_, extracted from the AUC data tempers these observations. While *f*/*f*_0_ is about 1.8 for FL-aSyn and pE24-aSyn, indicating an elongated shape, it is about 1.6 and 1.4, respectively, for pE62-aSyn and pE79-aSyn, suggesting shapes that are probably less elongated/more compact, in agreement with the change in intrinsic viscosity and viscosity increment.

Interestingly, AUC analysis showed the presence of higher-order species with higher-order oligomeric states. The aSyn variant p2 peaks detected by AUC were compatible with dimeric states of the respective variants. This was in agreement with the elution time, *R*_g_, *D*_max_, Kratky plots, as well as MW estimations (see Table S1) measured for FL-aSyn p2 by SEC-SAXS. To note, the radius of gyration, which is the root-mean-square distance to the center of mass of the particle, only increases by less than 3 Å between the monomer and dimer while the frictional coefficient decreases. These data point toward a side-by-side assembly or compaction as other configurations would have increased both *R*_g_ and frictional ratio. Finally, further peaks detected by AUC were compatible with different assemblies of the dimer such as tetramer, hexamer, or octamer as illustrated in Figure 6. The decrease in the frictional ratio values associated with these oligomers points to a more compact arrangement of the assembly rather than a possible elongation. This observation is compatible with peak p3 detected by SAXS, which corresponds to a higher oligomeric state with a more compact structure.

### SAXS: Ensemble Modeling of Monomeric and Dimeric States of FL-aSyn and pE-aSyn Variants

To gain further insight into the nature of all monomeric and dimeric states of FL-aSyn and pE-aSyn variants isolated in SEC, an ensemble analysis was performed by the Ensemble Optimization Method (EOM, ATSAS package) based on SAXS profiles (Figure 5).

**Figure 5:**
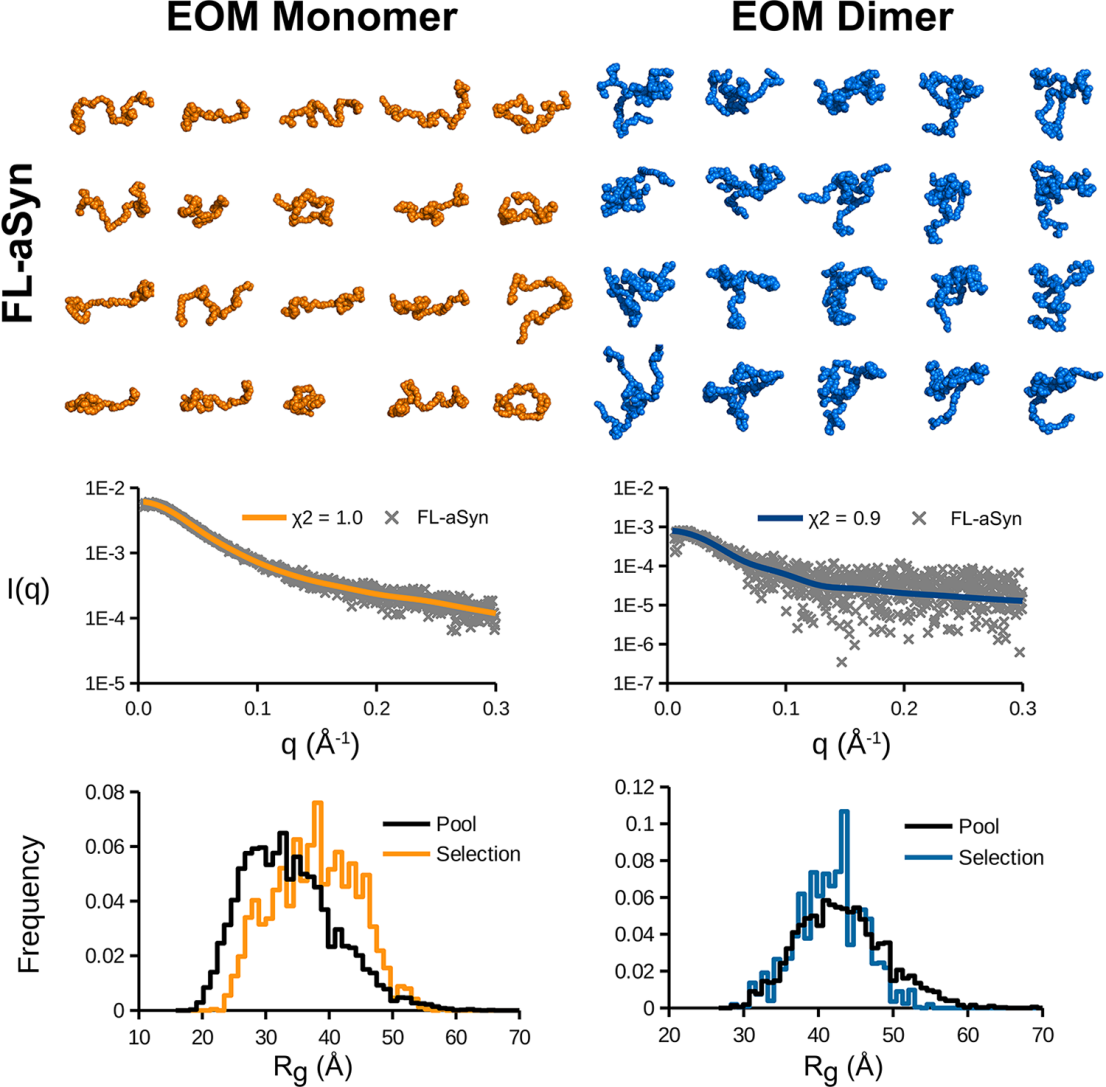
Ensemble modeling of FL-aSyn monomers and dimers performed using EOM 3.0 suite. Pools of 10,000 atomic models in random conformations were generated with RANCH and ensembles of 20 models were selected with GAJOE to reproduce the experimental data. The ensembles leading to the best fit (lowest *Chi* ^2^) for the monomeric (left panel) and dimeric (right panel) populations are depicted, as well as corresponding theoretical SAXS curves superimposed to the experimental data in the bottom panel, and the *R*_g_ distribution for the selected ensemble compared to the starting pool. All models are represented on the same scale.

Therefore, EOM 3.0 was first used to generate pools of 10,000 random models of FL-aSyn monomers and dimers. No secondary structure elements were imposed on the monomers, while the dimers included a central β-strand between aa residues 64–71 surrounded by floppy extremities, based on a recent model proposed by Savva & Platts (2024) in which two monomers are linked by short β-strands involving the region of aa 60–70. An ensemble of models was then selected from the starting pool, which collectively fit the experimental data best (Figure 5). The resulting ensembles give a good description of the possible conformational diversity of each type of particle. Monomeric models fit well with the idea of extended and flexible IDPs in solution, a feature confirmed by an *R*_g_ distribution centered on a higher value for the selection compared to the initial pool. As dimeric models present a constriction zone at the center that bridges the two monomers, their conformations are slightly more constrained and compact, leading to an *R*_g_ distribution closer to that of the original pool. Similar results were obtained for pE-aSyn monomers (Figure S2). In the absence of a starting model, dimeric forms of variants were not analyzed with EOM. Supporting data for Figure 5 and Figure S2 are available in the Supporting Information, containing models, experimental data, and the curves calculated from the corresponding ensembles.

Although less relevant for IDPs, GASBOR predictions were carried out with chained dummy atoms for both monomeric and dimeric forms (Figure S3), which gave similar qualitative results and, as expected, generated extended models. An attempt to model the particles present in peak p3 was performed using DAMMIF, since the Kratky plot indicated a compact structure (Figure S4). However, these populations are probably heterogeneous, as suggested by Awasthi *et al.* (2023).^59^ In addition, SAXS data are very noisy and the resulting models are reminiscent of those porous, fibril building blocks proposed by Giehm *et al.* (2011), which must be considered with caution.

### Bloomfield Models of the Assembly Step

In order to further understand the assembly intermediates such as dimer, tetramer and hexamer detected by AUC, a series of models compatible with the hydrodynamic results available (*S*, Mw, *R*_g_, *D*_max_) have been generated using a bead modeling assembly.^60^ In these hydrated models, aSyn monomers are represented as an array of spheres that has the overall shape of the FL-aSyn monomer EOM models, and the published structure of an aSyn(1–121) truncated monomer.^61^ The number of beads and their respective radius were chosen to occupy a volume compatible with the mass of the hydrated monomer. The hydrodynamic characteristics calculated from the FL-aSyn model presented in Figure 6 are in good agreement with the experimental values (see Table 1).

**Figure 6:**
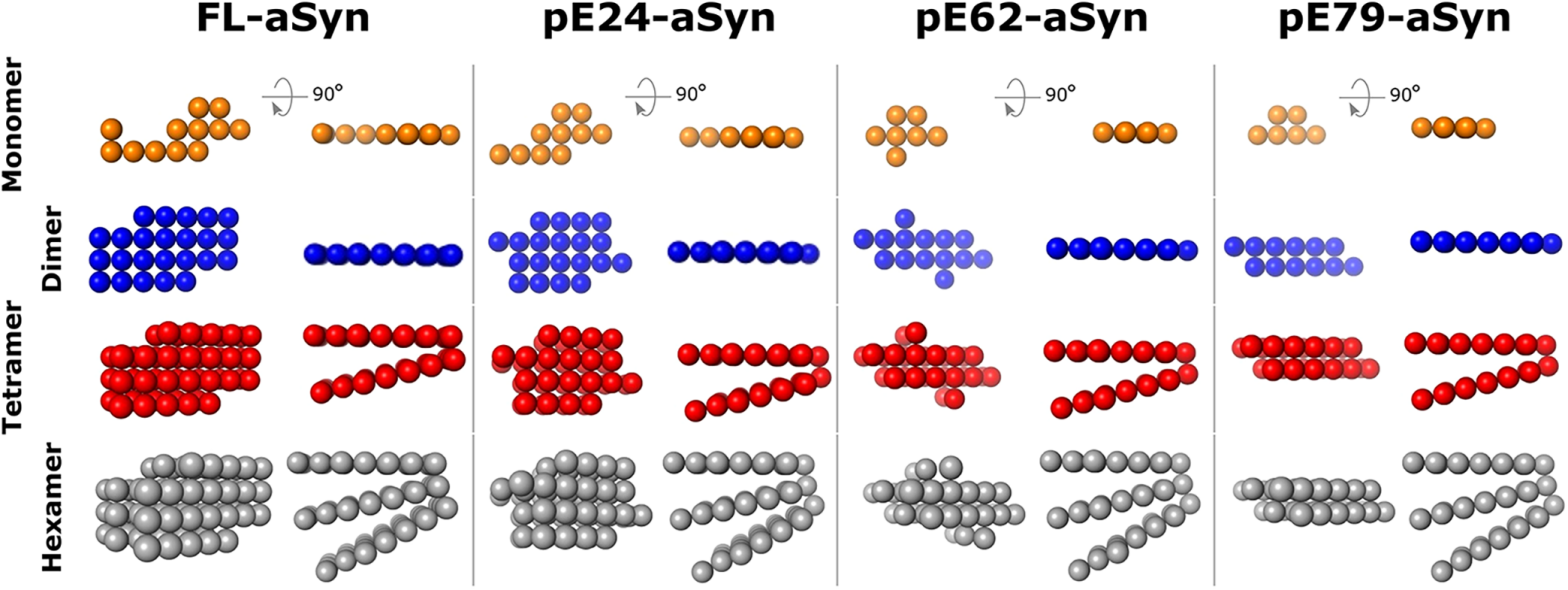
Hydrated bead models are compatible with hydrodynamic data. The models have been generated using a bead modeling assembly as proposed by ref 60. Their calculated hydrodynamic parameters are presented in Table 1 and are in agreement with experimental data.

This model of the FL-aSyn monomer was used to create a subsequent model for the dimer. Interestingly, SAXS measurements are in favor of a side-by-side arrangement of the monomers to form dimers whereas Cryo-EM data^61^ suggest a head-to-tail assembly. Consequently, the dimer model was created after a 180° turn of one of the monomers and then assuming side-to-side contact, resulting in compatible calculated hydrodynamic characteristics compared with hydrodynamic experimental data (see Table 1). For further elongation, this FL-aSyn dimer was then used as a building block to create a stacking assembly. To account for the spacing between dimers described in the cryo-EM structure (∼5 Å) and possible flexibility, the dimers were stacked at a 15° angle. All sedimentation coefficients calculated for oligomeric intermediates, tetramer, and hexamer, were compatible with the experimental data (see Table 1). Overall, this shows that all of the models are in favor of an elongation by stacking of the dimeric unit (see Figure 6).

Using this FL-aSyn model, pE-aSyn variants were generated by the removal of beads as a function of the expected hydrated volume of the different monomers. For all of them, the calculated sedimentation characteristics were compatible with the experimental data, providing evidence that the previously suggested assembly model could be applied to all pE-aSyn variants (see Figure 6).

### Toxicity of FL-aSyn and pE-aSyn Oligomeric States

In order to study the toxicity of oligomeric FL-aSyn and the pE-aSyn variants, in particular, pE24-aSyn and pE62-aSyn, a WST-1 assay was performed using differentiated SH-SY5Y neuroblastoma cells. Comparable experiments were previously carried out for pE79-aSyn.^49^ For oligomer preparation for cell culture experiments, agitated samples of FL-aSyn, pE24-aSyn or pE62-aSyn were taken from the respective lag phase of fibril formation. In Figure 7a, the statistical analysis of the lag phase periods shows shorter lag phases with increased truncation of the proteins. Thus, the lag time of fibril formation decreases from an average of 17 h for FL-aSyn to 6 h for pE24-aSyn and to 53 min for pE62-aSyn, confirming the instant fibril formation of pE62-aSyn reported above.

**Figure 7:**
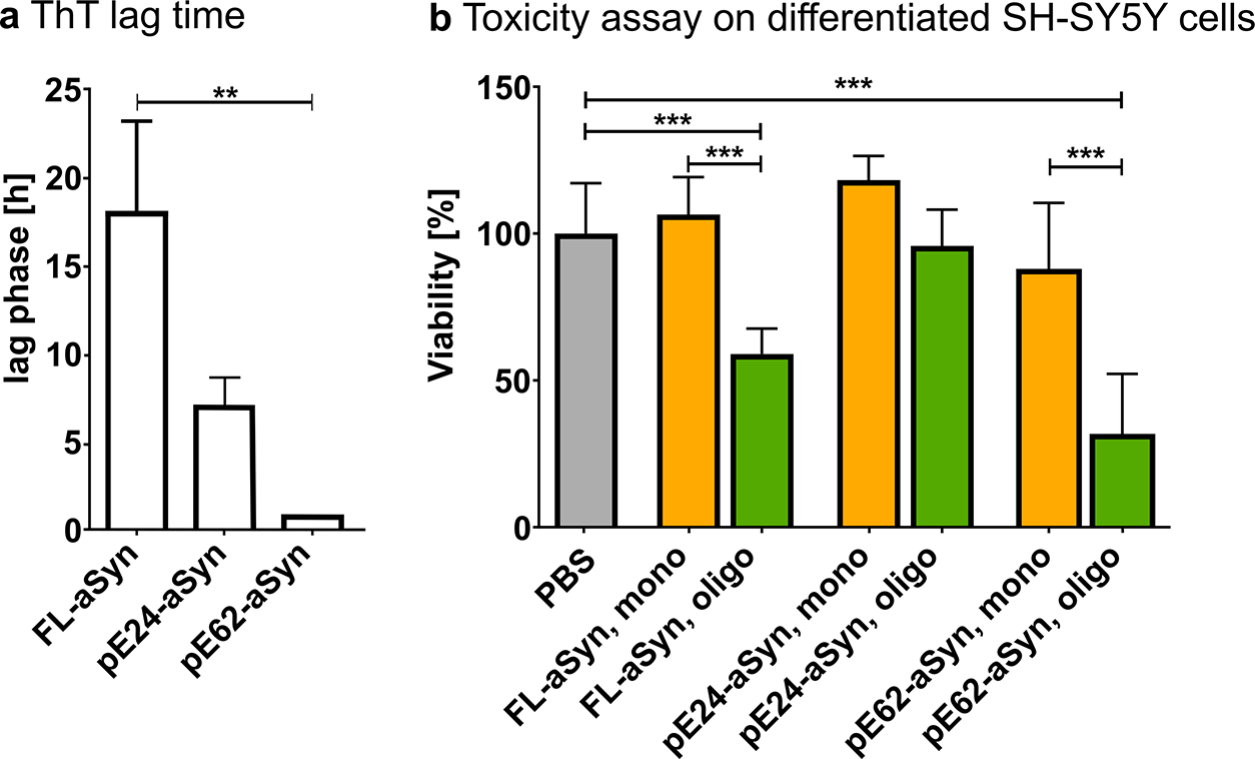
ThT lag times and cytotoxicity analysis of monomeric and oligomeric FL-, pE24-, and pE62-aSyn. (a) Statistical analysis of the lag phase of the aggregated FL-, pE24-, and pE62-aSyn (55 μM each). The analysis is based on a one-way analysis of variance (ANOVA) followed by a posthoc Tukey analysis (mean ± SD, *n* = 6; ** *p* < 0.01). (b) Cell viability was determined after 72 h of treatment with the respective variants in monomeric and oligomeric states (5 μM each) in differentiated SH-SY5Y cells using the WST-1 assay (mean ± SD, *n* = 3, ***p < 0.001 defined by one-way ANOVA followed by Tukey posthoc analysis).

For the toxicity assays, purified FL-aSyn, pE24-aSyn, or pE62-aSyn monomers and oligomers were applied on differentiated SH-SY5Y cells at a concentration of 5 μM each and normalized to the vehicle control PBS. Compared to the monomeric proteins, significant cytotoxicity of oligomeric aggregates of FL-aSyn and pE62-aSyn was observed, whereas oligomeric forms of pE24-aSyn only showed a tendency of reduced viability (FL-aSyn monomers: 107% viability vs oligomers 59% viability, pE24-aSyn monomers: 112% viability vs oligomers 96% viability, pE62-aSyn monomers: 87% viability vs oligomers 31% viability, see Figure 7b). In line with this, it has been previously shown that oligomeric pE79-aSyn induced significant cytotoxicity compared to its monomeric state (pE79-aSyn monomers: 73% viability, pE79-aSyn aggregates: 50% viability, for further details, see ref 49).

## Discussion

In this study, the structural characteristics of monomeric, dimeric, and oligomeric states of pE-modified aSyn variants were investigated in comparison to FL-aSyn. Due to the nature of the IDP aSyn, including the lack of a well-defined structure in the native state and high flexibility, studies of the structural properties are challenging. Therefore, an integrative biophysical approach, including DLS, SEC-MALS, SRCD, SAXS, and AUC was chosen to accomplish this goal.

This study was made possible by the use of standardized oligomerization protocols that improved the comparability of the results across different analytical methods. The investigation of aSyn oligomeric structures, with special focus on the initial assembly step – the dimeric state – has gained interest in recent years, as aSyn oligomers are classified as toxic and could serve as targets for therapeutic approaches.^7^ Standardized protocols for the generation of such aSyn oligomers are essential for data validity and the comparability of results. Such a detailed and reproducible oligomerization protocol was established by Paswalski *et al.* (2016) and thus served as the basis of this work.

### Unstructured and Elongated Monomeric FL-aSyn and pE-aSyn Variants

The orthogonal approach of analysis by SEC-MALS, SRCD, SAXS, and AUC provided detailed information on the secondary structure content, the overall size, and low-resolution shape of the monomeric FL-aSyn and pE-aSyn variants. The viscosity increments and the different elution times of the prominent peak p1 on SEC reflected the different lengths of the monomeric truncated pE-aSyn variants compared to FL-aSyn. Additionally, *R*_g_ values resulting from the SAXS curves were calculated for the monomers and the *R*_g_ value for the FL-aSyn monomer is in agreement with previously published data.^62,63^ Interestingly, the *R*_g_ value was smaller than expected for a completely unfolded polypeptide with a comparable number of amino acids (52 Å) but larger than the theoretical *R*_g_ of a 140-amino acid globular protein (15.1 Å), reflecting a possible folding or compression of the chain.^62,64^

Consistently, the shorter the length of the pE-aSyn variants, the smaller the respective *R*_g_ values became (35.0 Å for pE24-aSyn, 27.9 Å for pE62-aSyn, and 24.7 Å for pE79-aSyn). This relationship is reflected by the decreasing *D*_max_ values for FL-aSyn from 145 Å down to 104 Å for pE79-aSyn.

The hyperbolic Kratky curves of all analyzed aSyn variants confirmed the intrinsically disordered nature of aSyn. In addition, EOM-generated models for elongated monomeric states were proposed here for FL-aSyn and, for the first time, also for pE-aSyn variants. Other characteristic features of unfolded proteins were observed in the SRCD spectra with a strong negative peak near 200 nm and weak negative values at 220 nm.^54^ The lack of a defined secondary structure^9,10^ was reproduced for FL-aSyn as well as for all pE-aSyn variants at 27 °C indicating that the N-terminal truncation and pE modification does not affect the intrinsic disorder of the truncated aSyn polypeptides. The calculated β-strand content is stable across variants, reflecting the preferred transient β-strand conformation of the C-terminal residues 101–140,^62^ which is present in all aSyn variants investigated here. When the pE-aSyn variants were heated to 97 °C, a reduction in the negative 198 nm peak was observed, including a shift to 202 nm and an increase in the 222 nm band. A fully reversible pattern for heating and cooling was observed, indicating a conformational plasticity with reversibility following temperature increase,^65^ except for pE62-aSyn. For all aSyn variants analyzed, again with the exception of pE62-aSyn, an isosbestic point was observed, indicating that the stoichiometry remained unchanged and that no secondary reactions occurred during the temperature change.^66^ The data obtained for pE62-aSyn including the shortest ThT lag phase time, combined with the observation that upon heating the sample changed color from transparent to opaque in the cuvette, are indicative of fibrillation and highlight the fast aggregation propensity of this particular aSyn variant. Interestingly, a previous study also reported a high fibrillation propensity for the almost identical truncated—albeit unmodified—form of aSyn(61–140).^67^ The exact physiological function and toxicity of the monomeric pE-aSyn variants need to be further investigated in detail.

### SDS Micelles Forced FL-aSyn and pE-aSyn Variants into α-Helical Structure

To investigate potential conformational changes of the pE-aSyn variants upon lipid binding, a commonly used membrane-mimetic reagent, SDS, was chosen due to its ability to form micelles.^56,68,69^ The respective aSyn proteins folded immediately upon the addition of SDS paralleled by a significant increase in α-helical content, and the shorter the pE-aSyn variants, the lower the α-helical content. While the α-helical content of FL-aSyn increased by 37%, the α-helical content of the shortest pE79-aSyn variant increased by only ∼4%. This can be explained by the results of an NMR study, which showed that the 1–99 N-terminal aSyn region interacts with the SDS micelles.^70^ The transition to α-helical conformation is described as a broken α-helix with an antiparallel helical conformation (from aa ∼3–37 and aa ∼45–92) when bound to SDS micelles.^62^ In contrast, in the presence of small laminar vesicles (with a larger diameter compared to SDS micelles), aSyn adopts a continuous, extended α-helical conformation.^71^ It would be interesting to investigate the influence of aSyn truncations and pE-modifications on α-helical adaptations in the presence of small laminar vesicles to gain more detailed insight into aSyn-membrane interactions in physiological and pathological conditions. However, lipid binding associated with an α-helical conformation of aSyn inhibits its fibril formation.^69,70^ This is highlighted by the pE79-aSyn variant, which lacks most of the aa implicated in α-helix formation due to truncation, thus limiting the α-helical conformation. Within the amino acid sequence of the pE79-aSyn variant, the residues for transient β-strand conformation are present (101–140), potentially allowing oligomerization to occur.^62^ For all aSyn variants analyzed, except of pE79-aSyn, an isosbestic point was observed, indicating that the stoichiometry of secondary structure elements remained unchanged during the temperature change.^66^

### Dimeric FL-aSyn and pE-aSyn Variants—the Smallest Oligomer

To the best of our knowledge, for the first time, a SAXS-data based model of the dimeric state of FL-aSyn is proposed. The integrative approach combining AUC and upstream SEC prior to SAXS analysis allowed the separation of different aggregation states of FL-aSyn and of pE-aSyn variants, respectively. Other studies have also been able to detect the dimeric peak of aSyn in SEC, but either combined the data collection with the monomeric fraction^72^ or focused on the overall shape of the oligomers.^73^ Here, the separation of peaks and the analysis of their SAXS curves indicated an *R*_g_ for the FL-aSyn dimer of 39.5 Å with a *D*_max_ of 154 Å, thereby confirming simulated *R*_g_ values with 50.3 Å for aSyn dimers of Savva and Platts (2024). Compared with the experimental *R*_g_ of 36.7 Å and *D*_max_ of 145 Å for the FL-aSyn monomer, the size of the dimer did not increase significantly. Moreover, the frictional ratio decreased significantly from monomer to dimer from 1.8 to 1.6. The comparative analysis of these values indicates the existence of an intermediate state for the dimer between the mostly unfolded and folded states.

Interestingly, the observation of predominantly unstructured aSyn dimers with partial local structures was also reported by others.^74^ A more detailed analysis of the aSyn dimer conformation was recently performed by computational simulation under consideration of two aSyn monomers, so-called chains, showing intrachain β-hairpins consistent with antiparallel β-strands between residues V63-T72 in the NAC region (ChainA: T64-V71 and ChainB: V63-A69). Between the two hairpins, an interchain interaction was observed.^75^ Transferred to the shape of dimeric FL-aSyn described here, this interchain interaction may represent the connecting link to build a head-to-tail assembly of the monomer. The EOM modeling, based on this hypothesis of a short β-strand connector, highlights a transition from an extended, flexible, and unstructured monomer to a more compact dimer, which thereby is the smallest oligomer known for FL-aSyn.^76^

For pE62-aSyn, an accelerated fibrillation rate was described here. However, despite reducing the experimental aggregation time for pE62-aSyn from 5 to 0.5 h, neither a dimeric nor an oligomeric fraction was detected in addition to the monomeric fraction in the SEC chromatogram (see Figure 4a), while contradictory additional peaks were detected by AUC. The aSyn truncation at position Q62 and thus the N-terminal part of the pE62-aSyn variant is directly located at the beginning of the NAC region close to position V63-T72, where the β-hairpin and interchain interactions can be formed. Through the loss of steric hindrance of the N-terminus by truncation, a gain of aggregation-promoting properties with regard to dimerization/fibrillation can be hypothesized. These results are supported by the assumption of a protective role of the aSyn N-terminus against fibrillation.^67^

For pE79-aSyn, a dimeric state was detected by SAXS analysis but not by AUC. Nevertheless, it would be interesting to analyze the overall shape of this variant in future studies, as the conformers and interchain interactions described for FL-aSyn would not be applicable to this variant due to the truncation and partially missing NAC domain, so other forms of interactions might be implicated in dimer formation.

### aSyn Dimers as a Potential Therapeutic Target or as a Biomarker

The formation of dimers represents a potentially initial and crucial step in aSyn aggregation to toxic oligomers or fibrils. A recent finding underscoring this, is the 5-fold higher binding affinity of aSyn dimers for aSyn fibril ends compared to monomeric aSyn.^77^ Thus, alterations in the formation, stability, or dissociation of the dimers can influence the overall dynamics of aSyn aggregation. For example, an *in silico* study on graphene quantum dots accurately indicated a possible inhibition of aSyn dimerization by disrupting interchain interactions, leading to a looser and more unstable oligomeric conformation.^78^ Another therapeutic approach has been developed with covalent aSyn dimers, exploiting the higher binding affinity of the dimers at fibril ends. These covalent aSyn dimers were designed to bind fibril ends but block further growth, thereby slowing down the progression of PD.^77^ In addition, nonpeptide oxazole-based prolyl oligopeptidase inhibitors have been shown to reduce aSyn dimerization. When applied in *in vivo* studies in mice with aSyn pathology, motor impairment was reversed and levels of oligomeric aSyn were reduced in specific brain regions.^79^ Furthermore, the relevance of the aSyn dimer was also highlighted by the work of Papagiannakis *et al.* (2018), where the amount of aSyn dimers and the dimer/monomer ratio in erythrocyte membranes were suggested as a diagnostic tool for patients with genetic and nongenetic forms of PD.^80^ Taken together, aSyn dimers may serve as a new target for future therapies and biomarkers in PD research.

### Higher Oligomeric States of FL-aSyn and pE-aSyn Variants

The described aSyn dimer is the starting unit for further oligomerization as suggested by AUC (see Figure 6) resulting in the stacking assembly comparable to the previously published structure.^61^ AUC analysis did not allow for the detection of larger aggregates as most of them had sedimented during the acceleration phase of the experiment, however, they could be explored to some extent using SAXS. The *R*_g_ values for FL-aSyn, pE24-aSyn, and pE79-aSyn were of 60–64 Å and *D*_max_ of 173–220 Å and the corresponding Kratky plots clearly indicate that this oligomeric state exhibit folding and a more compact shape.

In line with this, the *ab initio* modeled FL-aSyn oligomer (see Figure S4) showed the shape of an annular structure, comparable to the shape of the oligomer of pE24-aSyn, which resembles a corkscrew. These results are consistent with the low-resolution SAXS model of Giehm *et al.* (2011), where a high protein concentration similar to this study was used to determine a so-called “wreath-shaped oligomer” with *D*_max_ of 180 ± 30 Å.^72^ Furthermore, those results agree with the observation of morphological studies employing high-resolution atomic force microscopy (AFM) and electron microscopy (EM), which indicated pore-like structures of oligomeric aSyn. While using AFM, an outer diameter of an aSyn pore of 160 Å was calculated^81^ whereas EM revealed an average diameter of 100–120 Å of a porous structure derived from a mutated form of aSyn (A30P or A53P).^73^ All of these descriptions of distinct oligomeric states might however only be considered as snapshots of a whole array of possible heterogeneous mixtures of species.^59,82^

The estimated number of monomers per oligomer may vary, depending on the preparation protocol. Previously published data suggested ∼16 monomers by SAXS analysis,^72^ 20–25 monomers by gel filtration,^83^ 14–50 monomers by fluorescence intensity distribution analyses,^84^ ∼20 monomers by fluorescence correlation spectroscopy^85^ and 11–39 monomers by nanopore-based, single-particle characterization, respectively.^59^

For FL-aSyn, pE62-aSyn, and pE79-aSyn^49^ significant cytotoxic potential of oligomeric aggregates, and for pE24-aSyn a tendency of reduced cell viability was observed in differentiated neuronal SH-SY5Y cells (see Figure 7b and for pE79-aSyn^49^). It is hypothesized, that the central channel of these oligomers has the potential to form a pore that can permeabilize the membrane.^86,87^ This membrane permeabilization was confirmed by *in vitro* experiments showing liposome disruption by the purified aSyn oligomers.^88^ It can be hypothesized that the data analyzed here correspond to the described amyloid pore and underline a possible formation of toxic porous structures by the oligomeric states of aSyn.

## Conclusions

The characterization of the biophysical properties of aSyn self-assembly intermediates is essential for a better understanding of the structure–toxicity relationship. Our integrative strategy provided a series of observations highlighting a variety of different structural characteristics among the FL-aSyn and pE-aSyn variants. SRCD performed at temperatures ranging from 27 to 97 °C revealed (i) the intrinsically disordered nature and (ii) the reversibility of the heating process for all variants in solution, except for pE62-aSyn, since the latter variant rapidly evolved toward fibrillation. This instant fibril formation, occurring even after short agitation time, may contribute to the toxicity and seeding properties of pE62-aSyn compared to FL-aSyn and analysis of fibril characteristics should be included in future studies. Furthermore, all aSyn variants investigated, except pE79-aSyn, exhibited a capacity to form α-helical content upon interaction with SDS micelles mimicking a membrane environment. Apparently, the phenomenon was dependent on the length of the (pE)-aSyn variant, with the shortest variant, pE79-aSyn, showing significantly less α-helical content. This impaired structural adaptation in the presence of membrane-like structures might indicate a loss-of-function of these pE-modified aSyn forms as compared to FL-aSyn in physiological processes such as presynaptic vesicle fusion.

The formation of aSyn dimers is very likely to represent the initial and crucial step of oligomerization and further aggregation/fibrillation. To the best of our knowledge, these are the first structural data on FL-aSyn and pE-aSyn dimers obtained by SAXS. The analysis of larger oligomeric shapes proved to be more challenging due to the low concentration of these populations, their heterogeneity, and the resulting weak signal-to-noise ratio. Nevertheless, the *ab initio* modeling of these oligomers observed for FL-aSyn and two pE-aSyn variants seems consistent with previous SAXS analysis. This, in combination with the cytotoxic potential of oligomeric (pE)-aSyn states, underlines the amyloid pore hypothesis of aSyn oligomers.

## Materials and Methods

### Expression and Purification of aSyn Proteins

In order to prepare the respective recombinant FL- and pE-modified aSyn proteins (Figure 8a), these aSyn variants were expressed in Escherichia coli employing four different pGEX-4T-1 vectors encoding the gene of human FL-aSyn, Q24-140-aSyn, Q62-140-aSyn, or Q79-140-aSyn proteins, fused in 5′ with a sequence coding for His_6_ and GST tags for protein purification and TEV protease cleavage site, to allow for N-terminal Q generation (Figure 8b).

**Figure 8:**
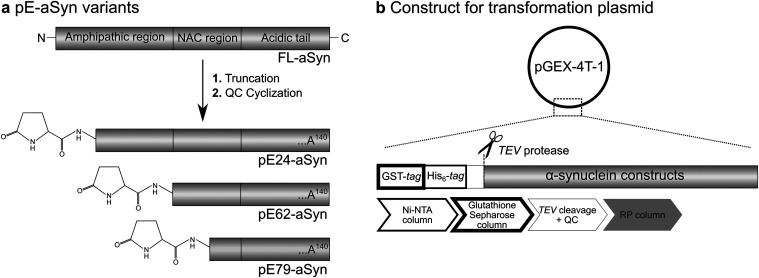
Schematic representation of (pE)-aSyn variants of interest and simplified depiction of the transformation plasmid for expression and purification of recombinant proteins. (a) Generation of FL-aSyn and pE-aSyn variants by truncation followed by QC-catalyzed pE cyclization resulting in pE24-aSyn, pE62-aSyn, and pE79-aSyn. (b) FL-aSyn as well as pE-modified aSyn variants are expressed in E. coli from pGEX-4T-1 vectors encoding Q24-aSyn, Q62-aSyn, and Q79-aSyn fused to a TEV protease cleavage site (to allow for N-terminal Q generation) and to His_6_ and GST tags for protein purification. Proteins expressed were first purified on a Ni-NTA column, followed by a glutathione sepharose column. Subsequently proteins were cleaved by TEV protease, incubated with QC to catalyze pE formation, and finally purified on a RP column. GST: glutathione-S-transferase; NTA: nickel-nitrilotriacetic acid; TEV: tobacco etch virus; QC: glutaminyl cyclase; RP: reversed phase; His_6_: polyhistidine.

The recombinant expression was described in detail previously.^89^ The first step of purification included Ni^2+‑^chelating chromatography on Streamline Chelating resin (Streamline Chelating, GE Healthcare Life Sciences, Uppsala, Sweden). Fractions containing the expression construct were further purified via glutathione sepharose resin (Glutathione Sepharose 4FF, GE Healthcare Life Sciences). The removal of the glutathione residue was performed by overnight dialysis against a buffer containing 100 mM NaCl, 30 mM Tris/HCl pH 7.6, 0.1 mM DTT, and using a 6–8 kDa cutoff membrane. To obtain a native N-terminus, the GST- and His-tag were separated from the aSyn by TEV protease cleavage.^90^ Subsequent cyclization of the Q-aSyn variants to pE-aSyn was achieved by incubation with QC (1 μg human QC per 1 mg peptide) overnight at room temperature, resulting in pE24-aSyn, pE62-aSyn, and pE79-aSyn (Figure 8). The reaction products were subjected to reverse phase chromatography (Source 15 RPC, GE Healthcare Life Sciences), followed by lyophilization and anion exchange chromatography (MonoQ 5/50GL, GE Healthcare Life Sciences). The purity of the samples was assessed by SDS PAGE and mass spectrometry (not shown). Protein concentrations of FL-aSyn, pE24-aSyn, pE62-aSyn, and pE79-aSyn were determined using UV absorption at 280 nm. Finally, the lyophilized protein variants were stored at −20 °C.

### Aggregation of Recombinant FL-aSyn and pE-aSyn Proteins

To ensure a seedless starting point at *t* = 0, samples were treated with 1,1,1,3,3,3-hexafluoro-2-propanol and were aliquoted prior to aggregation.^91^ For SAXS and AUC analysis, aSyn samples were prepared according to the modified aggregation protocol of Paslawski *et al.* (2016).^92^ Specifically, aliquoted samples were resuspended in 20 mM Tris/HCl pH 7.2 buffer with 100 mM NaCl to a final concentration of 12 mg/mL and shaken at 900 rpm at 37 °C for 5 h (Grant Instruments PCMT Thermoshaker, ThermoFisher Scientific). The agitation time of pE62-aSyn was reduced to 30 min because of the instant fibril formation. For removal of insoluble, fibrillary protein aggregates, samples were centrifuged at 10,000*g*, 4 °C for 10 min. For further analysis, the supernatant was transferred to a new tube and stored at 4 °C for up to 7 days.

### DLS and Static Light Scattering (SLS)

Resuspended samples were ultracentrifuged at 120,000*g*, 4 °C for 30 min (S45A rotor in Sorvall Hitachi Discovery M150E micro ultracentrifuge). For each protein solution, 8 μL of the supernatant were transferred into Wyatt disposable plastic microcuvettes, and scattered light was collected at 20 °C using a DynaPro Nanostar I light scattering instrument (Wyatt Technology, Santa Barbara, CA, USA) equipped with a modular He–Ne laser (10 to 100 mW) emitting at λ = 659 nm. Five measurements, each composed of 5 acquisitions for 5 s, were recorded at 20 °C and processed using DYNAMICS, version 7.8.1.3. (Wyatt Technology). Autocorrelation functions were analyzed using the so-called regularization fitting algorithm in order to extract characteristic times (hence, translational diffusion coefficients Dt) and amplitudes of the components in protein solutions. Hydrodynamic radii *R*_H_ of the corresponding species were then calculated using the Stokes–Einstein equation and size distributions were reported along with the polydispersity of each peak. Solvent refractive index (*n* = 1.351) and dynamic viscosity (η = 1.463 cP) needed for the Stoke–Einstein calculations were estimated using Malvern Panalytical DLS software, version 8, and the protein particle increment of the refractive index (d*n*/d*C*) was assumed to be 0.185 mL/g.

### SEC-MALS

Resuspended samples were immediately centrifuged at 27,000*g*, 4 °C for 15 min and were filtered by a Whatman PVDF filter device (pore size 0.2 μm) before measurements. SEC was coupled to a triple detection (concentration detector: UV detector and refractometer; static light scattering at 7 and 90°; viscometer) on an Omnisec RESOLVE and REVEAL instrument (Malvern Panalytical). Running buffer (20 mM Tris/HCl pH 7.2 with 100 mM NaCl) was filtered through 0.2 nm filters (Filtermax TPP) before equilibration of the column and detectors. Proteins were injected on a Yarra SEC 3000 column (Phenomenex, Aschaffenburg, Germany) at 20 °C. Samples were eluted at 0.5 mL/min after injection of a 100 μL sample at 5 mg/mL. External calibration was done with bovine serum albumin (BSA) (Sigma ref A1900) using an injection of 10 μL at 18.3 mg/mL. The refractive index, static light scattering, and viscosity measurements were processed to determine the average molecular mass and the intrinsic viscosity using the OMNISEC V11.30 software (Malvern Panalytical, U.K.).

### SRCD

SRCD spectra were recorded on the DISCO beamline^93^ at SOLEIL synchrotron (Saint-Aubin, France). The experimental setup was calibrated for magnitude and polarization with a 5.9 mg/mL d-10-camphorsulfonic acid solution. Four μL of FL-aSyn and of respective pE-aSyn variant solutions around 12 mg/mL were transferred to a CaF2 cuvette with an optical path of 27 μm. Three spectra from 180 to 262 nm were recorded at temperatures ranging from 27 to 97 °C in increments of 5 °C.

An equal volume of 200 mM SDS solution was added to the samples, resulting in a final SDS concentration of 100 mM to mimic a membranous environment. Indeed, in the presence of 100 mM NaCl, the critical micellar concentration of SDS was estimated to be 2 mM,^94^ which would guarantee the presence of micelles in conditions described here similar to those utilized in a previous NMR study.^68^

Spectra were averaged, scaled, and normalized to molar ellipticities after solvent baseline subtraction using CDtoolX.^95^ A data cutoff at 180 nm was applied based on photomultiplier high-tension viability. Secondary structure prediction was estimated from SRCD spectra using Web server BESTSEL (v1.3.230210).^96^ Using BESTSEL, α-helices are distinguished in regular helices with a middle part of α-helices and distorted helices with two or two residues at the ends. For β-strands, four subcategories are defined: (i) parallel β-strand, and antiparallel β-strand of three different twists: (ii) left-hand twisted, (iii) relaxed (slightly right-hand twisted), and (iv) right-hand twisted, respectively. Further secondary structure elements are turns or unstructured regions.^96,97^

### SEC-SAXS

SEC-SAXS experiments were performed on the SWING beamline at the SOLEIL synchrotron (Saint-Aubin, France).^98^ The beam wavelength was λ = 1.033 Å. The Eiger X 4 M detector (Dectris) was positioned at a distance of 2000 mm from the sample with the direct beam off-centered. The resulting exploitable *q*-range was 0.005–0.5 Å^–1^, where the wave vector *q* = 4πsin (θ)/λ and 2θ is the scattering angle.

The SEC-SAXS setup is illustrated in Figure S1.^99^ Prior to analysis, agitated samples were diluted with SEC running buffer 50 mM Tris/HCl pH 7.2 buffer with 200 mM NaCl to a total volume of 110 μL and were filtered by a Whatman PVDF filter device (pore size 0.2 μm). In Table S1, the exact volume of protein solution loaded onto the Yarra SEC 3000 column (Phenomenex, Aschaffenburg, Germany) installed on an Agilent HPLC system (20 °C) is described. Proteins were eluted at a flow rate of 0.3 mL/min. The eluates were analyzed by SAXS in a continuous flow capillary cell with a frame duration of 2990 ms at intervals of 10 ms.

Data processing was performed using Foxtrot^98^ and data analysis was conducted with the ATSAS 4.0.1 and BioXTAS-RAW 2.3.0 ^100,101^ packages. Peak deconvolution and baseline correction were performed in BioXTAS-RAW when required. Radii of gyration (*R*_g_) were derived from Guinier approximation and used to estimate the molecular weight based on various approaches: the Porod volume (*V*_p_),^102^ volume-of-correlation (*V*_c_),^103^ as well as Bayesian inference with the molecular weight calculations (datmw Bayes).^104^ *R*_g_ and maximal dimension (*D*_max_) were also calculated using the indirect transform package GNOM,^105^ which provides the distance distribution function *p*(*r*) of the particle. All of the values derived from these analyses are shown in Table S1.

EOM 3.0 (Ensemble Optimization Method)^106,107^ was used to generate extended flexible atomic models of monomers and dimers of FL-aSyn and pE-aSyn variants (10,000 models of each) and to search for ensembles of 20–50 models that collectively reproduce corresponding SAXS curves. *Ab initio* modeling was performed with GASBOR for shape reconstruction by a chain-like ensemble of dummy residues (Figure S3) and DAMMIF (Dummy Atom Modeling Minimization Fast) for shape determination based on single phase dummy atom models (Figure S4).^108^ Series of 30 models were produced with either software and were aligned using DAMAVER^109^ to select the most representative model of each series for comparison with other aSyn variants. Models were visualized with PyMOL Molecular Graphics Systems (Version 3.0 Schrödinger, LLC).

### AUC

Comparable to SAXS analysis, agitated samples were diluted with SEC running buffer 50 mM Tris/HCl pH 7.2 buffer with 200 mM NaCl to a total volume of 110 μL prior to AUC measurements. An additional measurement was carried out with doubled concentration. FL-aSyn, pE24-aSyn, pE62-aSyn, and pE79-aSyn proteins were centrifuged at 42,000 rpm in an Optima AUC analytical ultracentrifuge (Beckman Coulter), at 20 °C using an 8-hole AN 50–Ti rotor equipped with 3 mm double-sector Epon charcoal center pieces. The concentrations of the individual macromolecules were chosen to be detectable by the AUC equipment without saturating the detectors. Detection of the biomolecule concentration as a function of the radial position and time was performed by interference detection. Sedimentation velocity data analysis was performed by continuous size distribution analysis c(s) using Sedfit 16.36 software.^110^ All of the c(s) distributions were calculated with a fitted fractional ratio *f*/*f*_0_ and a maximum entropy regularization procedure with a confidence level of 0.68. The detected peaks were integrated to obtain sedimentation values and the percentage of each detected species. Frictional ratios were calculated from the sedimentation coefficient and the known molecular mass by using the Svedberg equation. Buffer viscosity and density were calculated from Sednterp 3.0.4 software. Partial specific volumes and theoretical monomer molecular weight were also determined with Sednterp 3.0.4 (Table S2).

### Toxicity Assay of Monomeric and Oligomeric FL-aSyn and pE-aSyn Variants in Differentiated SH-SY5Y Neuroblastoma Cells

The potential cytotoxic effects of monomeric FL-aSyn, pE24-aSyn, and pE62-aSyn and their corresponding oligomers on SH-SY5Y neuroblastoma cells were assessed using a WST-1 assay (ThermoFisher, Darmstadt, Germany).

To generate FL-aSyn and pE-aSyn oligomers, an aggregation assay was performed as previously described.^111^ Recombinant FL-aSyn, pE24-aSyn, or pE62-aSyn was prepared for agitation to a final concentration of 250 μM in aggregation buffer (20 mM Tris/HCl, 100 mM NaCl, pH 7.0) and incubated with 20 μM ThT for monitoring fibril formation or without ThT in parallel for subsequent purification. Monomeric protein was incubated in a 96-well plate using a CLARIOstar plate reader (BMG Labtech) with continuous shaking at 300 rpm and 37 °C, and signals were recorded at 15-min intervals. The excitation and emission wavelengths were 440 and 490 nm, respectively. Analyses of the obtained aggregation curves were conducted according to Hortschansky *et al.* ^112^

Aggregated samples of FL-aSyn, pE24-aSyn, or pE62-aSyn were taken in the lag phase at different time points depending on the aggregation velocity (FL-aSyn: 7 h, pE24-aSyn: 7 h, and pE62-aSyn: 1 h; see Figure 7a) and purified by SEC. The samples were centrifuged at 17,000*g* for 12 min to remove large aggregated particles. The supernatants were diluted in PBS (pH 7.0) to a total volume of 250 μL, and subsequently loaded at a flow rate of 0.8 mL/min onto a Superdex 75 Increase 10/300 GL column on an ÄKTA avant 25 system (Cytiva, Freiburg, Germany). The eluted peaks were monitored at 220 and 280 nm.

SH-SY5Y cells were grown in Dulbecco’s modified Eagle’s medium (DMEM) medium supplemented with 10% fetal bovine serum (FBS) at 37 °C and 10% CO_2_. To induce differentiation toward a neuronal phenotype, 1.83 × 10^4^ cells/well were seeded in a transparent 96-well plate, and the medium was changed to DMEM supplemented with 5% FBS and 10 μM all-trans retinoic acid (ThermoFisher, Darmstadt, Germany) for 3 days. The medium was further exchanged to Neurobasal-A medium without phenol red, supplemented with 1% (v/v) Glutamax, 1% (v/v) N-2 supplement (ThermoFisher, Darmstadt, Germany), and human BDNF (ThermoFisher, Darmstadt, Germany) at a concentration of 50 ng/mL (v/v) for additional 4 days. On day 7 of differentiation, the assay was carried out according to the manufacturer’s protocol. In brief, the cells were exposed to different aSyn proteins and aggregation states and cultured at 37 °C in a humidified atmosphere containing 10% CO_2_ for 72 h. Afterward, 10% WST-1 was added to the cell medium and incubated for 30 min. The absorbance was determined at 440 nm using a plate reader (Tecan Sunrise, Switzerland). The values were normalized to the PBS control and directly correlated to the number of viable cells. Corresponding data for pE79-aSyn have been previously published and can be accessed at ref 49.

## Supplementary Material



## Data Availability

The data sets used and analyzed during the current study are available from the corresponding author on reasonable request.

## Notes

Principle of the SEC-SAXS setup (Figure S1); ensemble modeling of monomers of FL-aSyn and pE-aSyn variants performed using EOM 3.0 suite (Figure S2); *ab initio* reconstructions of monomers and dimers of FL-aSyn and pE-aSyn variants (Figure S3); *ab initio* modeling of oligomeric states of FL-aSyn and pE-aSyn variants based on SAXS data (Figure S4); structural parameters of SEC-SAXS data analysis (Table S1); Biophysical constants used to analyze AUC data (Table S2); Supporting data: EOM-aSyn-models.zip: archive containing model ensembles generated with EOM to fit the SAXS curves displayed in Figures 5 and S2, as well as spreadsheets with the experimental data and the curves computed from the corresponding ensembles (FLmono, FLdimer, pE24mono, pE62mono, pE79mono) in standard ODS format (PDF)

## ○

Shared senior authors.

## List of Abbreviations

aSyn: α-synuclein
AUC: analytical ultracentrifugation
pE: pyroglutamate
SEC: size exclusion chromatography
SAXS: small-angle X-ray scattering
SRCD: synchrotron radiation circular dichroism
