# Supplementary material for: Monomers, Dimers, and Oligomers of Pyroglutamate-Modified α‑Synuclein Fragments Exhibit Distinct Biophysical Characteristics
Source: ACS Chem Neurosci. 2025 Apr 30;16(10):1919–36. doi: 10.1021/acschemneuro.5c00106 (PMC12100652; doi:10.1021/acschemneuro.5c00106)
Supplement: Supplementary file 1 [file cn5c00106_si_001.pdf]

Supporting information:

Monomers, dimers and oligomers of pyroglutamate-modified alpha-synuclein fragments exhibit distinct biophysical characteristics

Alexandra Bluhm[1], Wei Xiang[2], Frank Wien[3], Aurelien Thureau[3], Maelenn Chevreuil[4], Bertrand Raynal[4], Stefanie Geissler[5], Michael Wermann[5], Stephan Schilling[5,6], Philippe Bénas[7], Maike Hartlage-Rübsamen[1], Anja Schulze[5,6], Claude Sauter[7]* and Steffen Roßner[1]**

1 Paul Flechsig Institute – Centre for Neuropathology and Brain Research, University of Leipzig, 04103 Leipzig, Germany

2 University Hospital Erlangen, Department Molecular Neurology, Friedrich-Alexander-University Erlangen-Nürnberg, 91054 Erlangen, Germany

3 Synchrotron SOLEIL, L'Orme des Merisiers Saint Aubin, 91410 Gif-sur-Yvette, France

4 Plateforme de biophysique moléculaire, C2RT, Institut Pasteur, Université Paris Cité, 75015 Paris, France

5 Fraunhofer Institute for Cell Therapy and Immunology, Department of Molecular Drug Design and Target Validation, 06120 Halle (Saale), Germany

6 Faculty of Applied Biosciences and Process Engineering, Anhalt University of Applied Sciences, 06366 Köthen, Germany

7 CNRS, Architecture et Réactivité de l'ARN, UPR 9002, Institut de Biologie Moléculaire et Cellulaire, Université de Strasbourg, 67084 Strasbourg, France

* *shared senior authors*

Corresponding authors:

Professor Steffen Roßner, PhD

Paul Flechsig Institute – Centre for Neuropathology and Brain Research

Liebigstraße 19

04103 Leipzig

GERMANY

Phone: +49-3419725758

Email: steffen.rossner@medizin.uni-leipzig.de

Dr. Claude Sauter, PhD

Institut de Biologie Moléculaire et Cellulaire

ARN – UPR 9002 – CNRS, Unistra

2, Allée Conrad Roentgen

67084 Strasbourg

FRANCE

Phone: +33-388417102

Email: c.sauter@ibmc-cnrs.unistra.fr

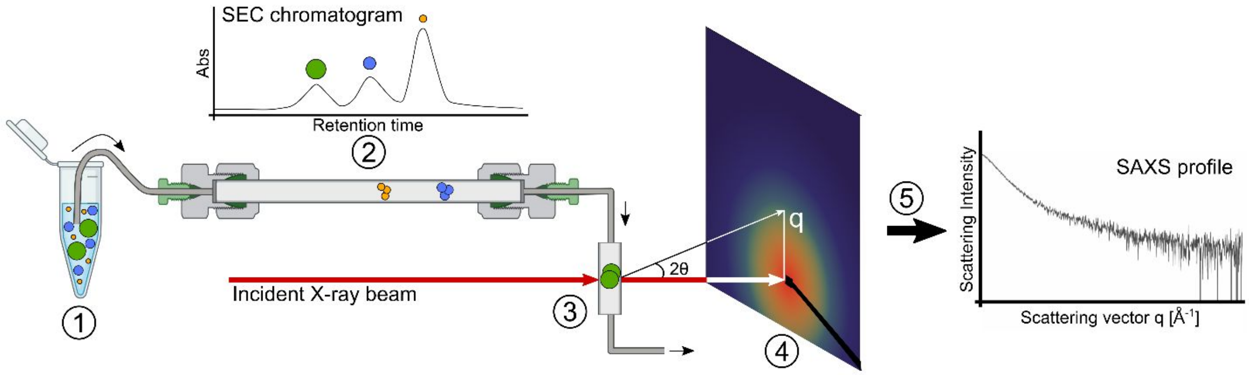

Figure S1: Principle of SEC-SAXS setup. Agitated FL-aSyn and pE-aSyn variants **(1)** were loaded onto a HPLC-SEC column **(2)** to separate particles by size. The eluates were directly injected in the SAXS capillary cell **(3)** and scattering patterns of X-rays were collected on a 2D detector **(4)** and processed by subtracting the solvent signal from the sample **(5)**. Structural information on the size and the shape of the scattering particle in solution were then calculated from the curve *I(q)* vs *q* (adapted from Théobald-Dietrich *et al*., (2020)). Created with BioRender.com.

EOM Monomers

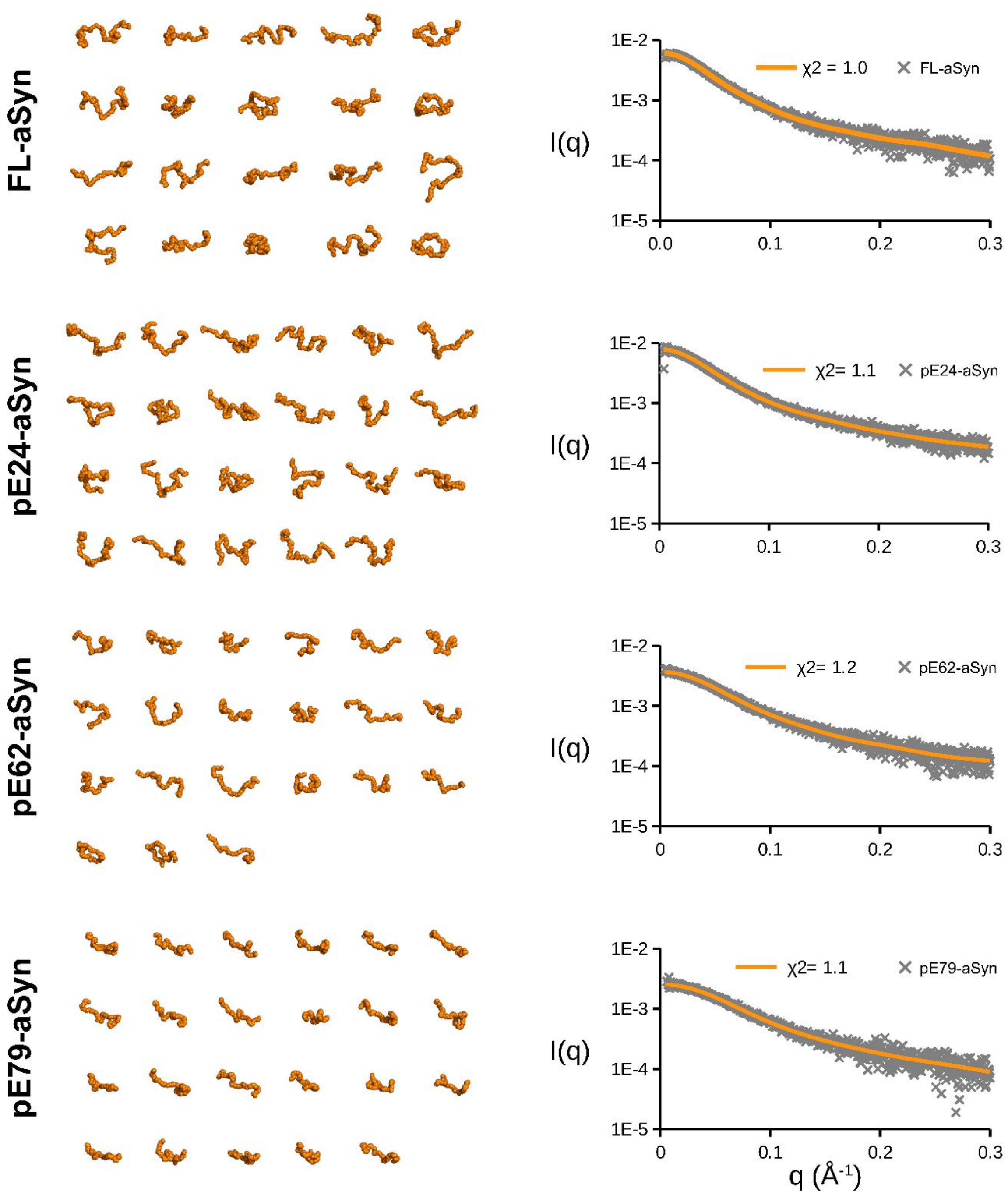

Figure S2: Ensemble modelling of monomers of FL-aSyn and pE-aSyn variants performed with EOM 3.0 suite. Pools of 10000 atomic models in random conformations were generated with RANCH and ensembles of ~20- models were selected with GAJOE to reproduce the experimental data. The ensembles leading to the best fit (lowest *Chi*^{2}) are depicted on the left, as well as corresponding theoretical SAXS curves superimposed to the experimental data on the right. All models are represented at the same scale.

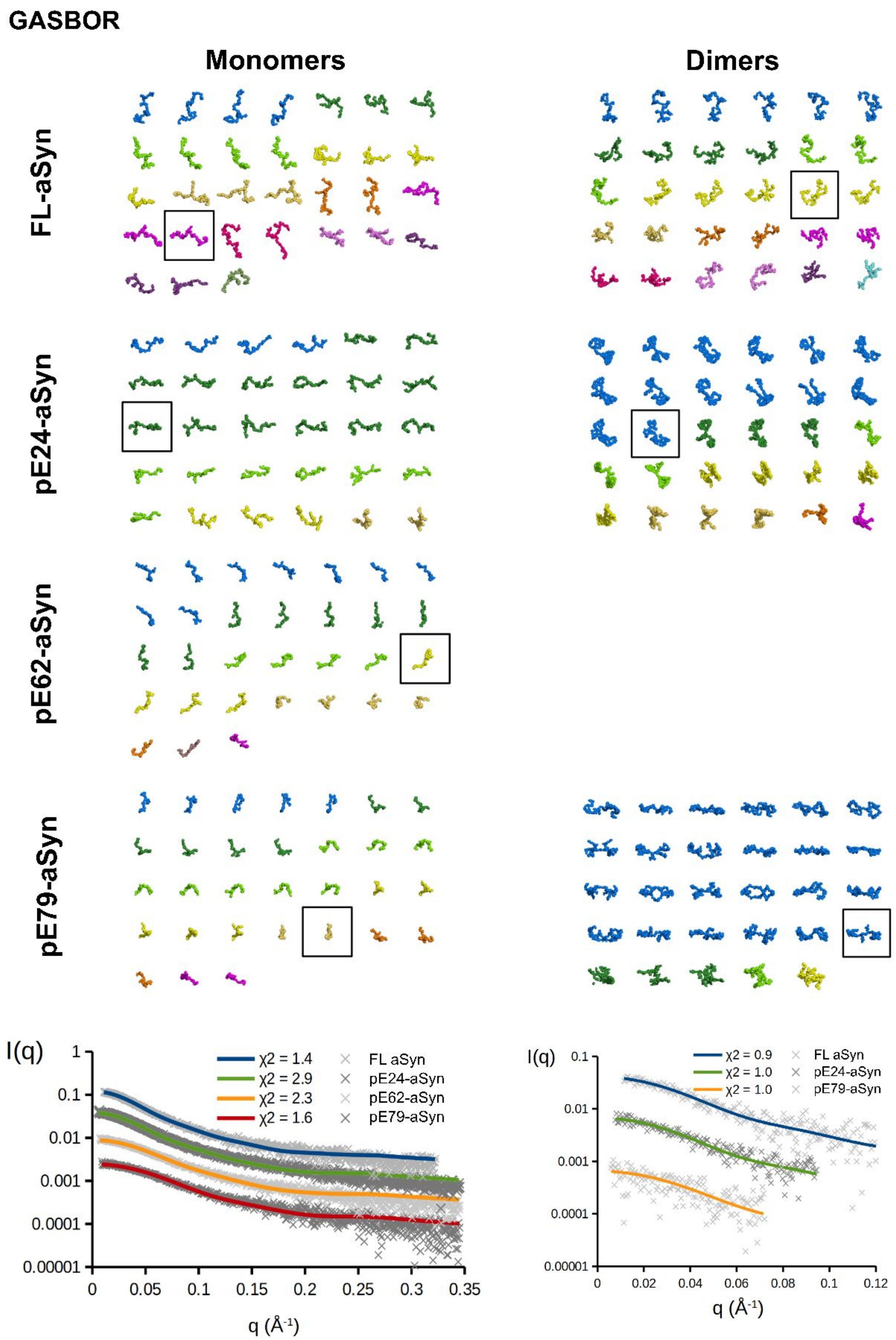

Figure S3: *Ab initio* reconstructions of monomers (left) and dimers (right) of FL-aSyn and pE-aSyn variants. Molecular shapes made of dummy residues or atoms were reconstructed with GASBOR, respectively. About 30 models were generated and aligned and clustered with DAMAVER. Models belonging to the same cluster are depicted with the same color. Boxed

models are the most representative in each series according to DAMAVER analysis. Their theoretical SAXS curves are superimposed to the experimental data and associated goodness of fit values are indicated in the bottom panel. All models are represented at the same scale.

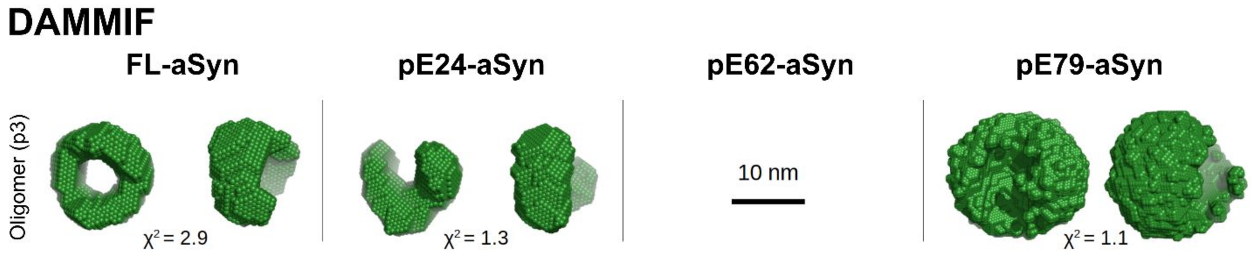

Figure S4: *Ab initio* modelling of oligomeric states of FL-aSyn and pE-aSyn variants based on SAXS data. Molecular shapes made of dummy atoms with DAMMIF (compact oligomers). 30 models were generated and the most representative model following DAMAVER superposition is displayed for each sample. Models of the different oligomeric states are represented at the same scale in two orientations, with the same color as their corresponding elution peaks and SAXS curves in Figure 4 (green). SAXS data of oligomers adopt a compact tor-like shape. Note that for pE62-aSyn only a monomeric fraction in SEC was detectable. All models are represented at the same scale.

Table S1: Structural parameters of SEC-SAXS data analysis.

			LCS analysis	BioXTAS RAW 2.3.0							ATSAS 4.0.1	GNOME		
	V_{inj} (µl)	SEC-Peaks	data range	R_g Guinier (Å)	q_max*R_g	Correlation coefficient, R^2	qR_g range (Å^{-1})	I(0) (cm^{-1})	datmw Bayes MW (kDa)	Vc-Vp MW (kDa)	MoW (kDa)	R_g P(R) (Å)	qR_g range (Å^{-1})	Dmax (Å)
**FL-aSyn**	85	p1	309-312	36.88 +-0.35	1.29	0.98	0.011-0.035	6.13E-03	16.8	22.7-37.5	16.5	39.84+-0.33	0.011-0.323	145
		p2	241-249	39.53 +- 2.49	1.29	0.68	0.011-0.032	8.10E-04	47.7	43.8-70.8	26.4	44.69+-1.43	0.011-0.095	154
		p3	108-123	64.16 +-4.55	1.29	0.69	0.008-0.020	1.39E-03	585	1.28E+03-728.9	N/A	62.30+-1.58	0.008-0.049	173
**pE24-aSyn**	75	p1	317-322	34.97 +- 0.22	1.29	0.98	0.006-0.037	7.85E-03	16.1	19.4-30	14.6	37.16 +- 0.19	0.006-0.345	135
		p2	248-261	48.13 +- 2.53	1.29	0.50	0.008-0.027	7.35E-04	58.1	54.3 +- 91.8	60.7	49.85+-1.39	0.008-0.094	166
		p3	114-122	60.69 +- 6.02	1.27	0.67	0.012-0.021	1.06E-03	585.2	808.8-589.6	N/A	64.59+-2.68	0.012-0.049	217
**pE62-aSyn**	86	p1	342-349	27.90 +- 0.22	1.29	0.97	0.007-0.046	3.67E-03	13.4	13.5-14.6	9.3	30.04 +- 0.29	0.012-0.345	115
**pE79-aSyn**	86	p1	352-357	24.68 +- 0.24	1.29	0.95	0.009-0.052	2.55E-03	10.4	11.6-10.3	8.1	26.43 +-0.31	0.009-0.345	104
		p2	295-305	39.81 +- 4.83	1.29	0	0.006-0.032	2.93 E-04	54.4	60.9-74.2	N/A	43.85 +- 2.99	0.006-0.072	145
		p3	106-117	62.63 +-5.41	1.29	0.22	0.011-0.020	4.63E-04	646.8	2.05E+03-N/A	N/A	72.72 +-3.92	0.011-0.049	224

Extended data **Table S2:** Biophysical constants used to analyze AUC data. The partial specific volumes of each protein were estimated from their aa sequences using the software Sednterp 3.0.4 (available online from The Boston Biomedical Research Institute). The same software was used to estimate buffer viscosity and density.

	Theoretical monomer MW (Da)	Partial specific volume ($\bar{v}_i$, ml/g)	Buffer density (ρ, g/ml)	Buffer viscosity (η, poise)
FL-aSyn	14,460	0.734 ± 0.001	1.00298	0.01017
pE24-aSyn	12,137	0.729 ± 0.001	1.00298	0.01017
pE62-aSyn	8,298	0.721 ± 0.001	1.00298	0.01017
pE79-aSyn	6,773	0.713 ± 0.001	1.00298	0.01017

Supporting data: EOM-aSyn-models.zip: archive containing model ensembles generated with EOM to fit the SAXS curves displayed in figure 5 and S2, as well as spreasheets with the experimental data and the curves computed from the corresponding ensembles (FLmono, FLdimer, pE24mono, pE62mono, pE79mono) in standard ODS format. PyMOL scripts are provided to facilitate the examination of these ensembles.
